# Persistent Cytotoxicity and Endocrine Activity in the First Oil Sands End-Pit Lake

**DOI:** 10.1021/acsestwater.2c00430

**Published:** 2023-01-24

**Authors:** Ian G. M. Gault, Chenxing Sun, Jonathan W. Martin

**Affiliations:** † Division of Analytical and Environmental Toxicology, University of Alberta, Edmonton, Alberta T6G 2G3, Canada; ‡ Department of Environmental Science, Stockholm University, Stockholm 106 91, Sweden

**Keywords:** oil sands process-affected water, extractable organics, cytotoxicity, real-time cell analysis, electrospray ultra-high-resolution mass spectrometry, endocrine activity

## Abstract

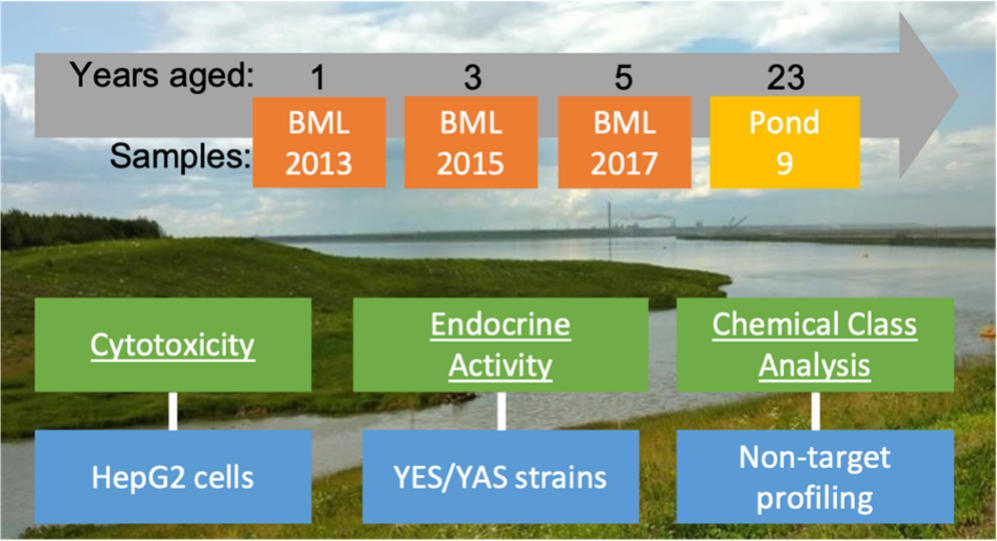

Oil sands process-affected water (OSPW) is a byproduct of bitumen extraction that has persistent toxicity owing to its complex mixture of organics. A prominent remediation strategy that involves aging OSPW in end-pit lakes and Base Mine Lake (BML) is the first full-scale test. Its effectiveness over the first 5 years was investigated here using real-time cell analysis, yeast estrogenic and androgenic screens (YES/YAS), and ultra-high-resolution mass spectrometry. HepG2 cytotoxicity per volume of BML organics extracted decreased with age; however, the toxic potency (i.e., toxicity per mass of extract) was not significantly different between years. This was consistent with mass spectral evidence showing no difference in chemical profiles, yet lower total abundance of organics in field-aged samples, suggestive that dilution explains the declining cytotoxicity in BML. The IC_50_’s of BML extracts for YES/YAS antagonism were at environmental concentrations and were similar despite differences in field-age. Persistent YES/YAS antagonism and cytotoxicity were detected in experimental pond OSPW field-aged >20 years, and while organic acids were depleted here, non-acid chemical classes were enriched compared to BML, suggesting these contribute to persistent toxicity of aged OSPW. To avoid a legacy of contaminated sites, active water treatment may be required to accelerate detoxification of end-pit lakes.

## Abstract 2

Persistent cytotoxicity and endocrine activity are observed for OSPW samples despite field-years aged. Dilution, not degradation, is the primary remediating factor.

## Introduction

Oil sands process-affected water (OSPW) is a byproduct of bitumen extraction in the surface-mining oil sands industry of Northern Alberta, Canada. OSPW contains a complex and environmentally persistent dissolved organic mixture that can be toxic to aquatic^1−3^ and mammalian^4,5^ species. A prominent long-term remediation strategy involves aging OSPW in end-pit lakes such that *in situ* natural processes will eventually allow detoxification and safe re-integration of this water to the regional watershed. Over 30 end-pit lakes are planned, but only Base Mine Lake (BML) has been established so far, commissioned in 2012 at Syncrude Canada Ltd. Concerns and uncertainties about end-pit lakes have been highlighted by the Royal Society of Canada,^6^ in particular that the rate of biodegradation for bitumen-derived organics is expected to be slow^7^ and that the detoxification rate at full-scale remains unknown.

The dissolved organic chemical mixture in OSPW is derived from bitumen during extraction and has been described as a supercomplex.^8,9^ Approximately 3000 distinct chemical species (i.e., distinct chemical formulas) are routinely detectable in fresh OSPW by high-pressure liquid chromatography (HPLC) with high-resolution mass spectrometry (HRMS) detection from both positive and negative ionization modes.^10^ Moreover, for each of these 3000 species, there may be hundreds or thousands of isomers that are generally not possible to resolve by the best available methods.^11,12^ In practice, bitumen-derived substances in OSPW are therefore profiled at the chemical species level by their empirical formula in both ionization modes, e.g., C*_x_*H*_y_*O*_z_*S_a_N_b_^+/–^, and more broadly by their heteroatomic formula class, e.g., O_3_^–^, O_2_^+^, SO^+^. Only chemical species belonging to the O_2_^–^ class (also termed naphthenic acids, NAs) have been studied with regard to environmental persistence under field conditions and have estimated disappearance half-lives in excess of 12 years;^7^ the persistence of all other chemical classes in OSPW remains unknown.

Pilot-level experimental ponds have been studied in the field over the past three decades at Syncrude Canada Ltd. to examine parameters that most effectively assist OSPW detoxification.^13^ One such study showed that OSPW aged 13–15 years still had chronic toxicity attributable to the dissolved bitumen-derived organics.^14^ Other studies of aged OSPW with fish^1−3,15^ and midges^16^ have reported reproductive and developmental effects, but that toxicity decreases with aging, coinciding with lower NA concentrations. Another study reported reduced testosterone and estradiol in both male and female goldfish exposed to OSPW in reclamation ponds for 17 days;^17^ explants of the gonadal tissues from males and females in this study had significantly lower basal levels of testosterone after OSPW exposure. In a follow-up study, goldfish were exposed in the lab for 7 days to a NA isolate from OSPW, but the results could not be reproduced,^17^ suggesting that chemicals other than NAs may be responsible for the endocrine effects.

When BML was first commissioned, the water underwent an effects-directed analysis to identify acutely toxic chemical classes, and these included the O_2_^–^ class as well as non-acid classes, including O^+^, O_2_^+^, SO^+^, and NO^+^.^18^ Subsequent monitoring of the toxicity in BML has largely been limited to industrial monitoring and associated reports to government regulators.^19^ Interpretation of these data by the industry has not yet revealed long-term trends in toxicity and has been complicated by active management of the water balance in the lake. Such management has included pumping out OSPW from the surface into the extraction process and dilution from pumping in freshwater, as well as from natural runoff and precipitation. Another dynamic in BML is the *in situ* dewatering of the underlying fine tailings, which as they naturally compact, releases pore water to the lake’s surface.^19^ White and Liber^20^ reported that the pore water expressed during fine tailings densification is similar to fresh OSPW and that this continual slow input into the overlying OSPW may counteract any slow degradation.

From *in vitro* toxicology studies, there are substantial evidence for endocrine activity in OSPW, including estrogen and androgen receptor (ER/AR) agonism and antagonism^21−23^ and altered steroidogenesis and estrogen (E2) metabolism.^22^ NAs are surfactants, leading to speculation by Frank et al.^24^ that, like other surfactants,^25^ NAs act through narcosis as the predominant mechanism of cytotoxic action. This idea is supported by more recent studies.^18,26^ Nevertheless, there is variability in published cytotoxicity results, with some studies showing a cytotoxic effect in fish cell lines^27^ or no effect on cell viability in fish hepatocytes^28^ or human cell lines,^22^ and even enhanced cell proliferation in mouse bone-derived macrophages.^29^ We hypothesized that some of this variability may be due to limitations of most cytotoxicity assays that measure an effect at only one time point in the assay.

To evaluate the effectiveness of natural aging of OSPW in end-pit lakes, the objectives of the current study were to comprehensively analyze cytotoxicity, endocrine activity, and chemical profiles of dissolved organics in OSPW of different field-ages, including from sequential samples from BML and another aged experimental pond containing OSPW. Cytotoxicity measurements were made in human cells by real-time cell analysis (RTCA), thereby allowing time-resolved toxicity profiles to be compared, while endocrine activity was measured in yeast, genetically modified to express the human estrogen or androgen receptors. The OSPW samples were also characterized by HRMS to examine for differences in organic chemical profiles as evidence for *in situ* degradation processes.

## Materials and Methods

### Sample Collection and Source

OSPW samples were provided by Syncrude Canada Ltd. and stored at 4 °C in high-density polyethylene pails. The active tailings pond, West In-Pit, was renamed as BML and commissioned as an end-pit lake in fall 2012. Thus, BML samples collected in the summer of 2013, 2015, and 2017 were considered to be field-aged 1, 3, and 5 years, respectively. The longer field-aged OSPW sample (23 years) was collected in 2016 from an experimental pond known as Pond 9, which was commissioned in 1993 and had an input of OSPW originating from the active tailings pond, Mildred Settling Basin.^7^ Differences in experiment outcomes may exist due to differences in OSPW source between BML and Pond 9; however, all of the experimental ponds have limitations to their BML comparisons in terms of composition, depth, and scale. Here, Pond 9 has been used as an indicator, out of a subset of potential experimental pond indicators, of what BML could be like in the future after an equivalent amount of aging. An Athabasca River water sample was collected in 2017 as an environmentally relevant natural reference, referred to subsequently as “River 2017,” from an upstream location (Figure S1) which is near the uppermost regions of the McMurray Geological Formation.^30^ It is known from our past work and previous public reports that OSPW seepage has impacted McLean Creek;^31,32^ therefore, River 2017 was approximately 6 km upstream of McLean Creek to ensure a representative freshwater sample. Sample collection methods are in the SI.

### Isolation of Extractable Organics

A liquid–liquid extraction was used to extract dissolved organics from samples at acidic pH. First, 1 L of the sample was vacuum-filtered with a G4 glass fiber filter and acidified to pH 2 with dropwise addition of concentrated sulfuric acid (98%) (Thermo Fisher Scientific, San Jose, CA). Extraction was performed in 2 L glass separatory funnels with 200 mL of dichloromethane (99.5%) and repeated three times. The combined extracts were concentrated by rotary evaporation (Rotavapor R-210, Buchi, Flawil, Switzerland), transferred to a preweighed glass vial, and brought to dryness by nitrogen evaporation (TurboVap LV, Caliper Life Sciences, Hopkinton, Massachusetts). The dry organic mass was recorded and reconstituted in 200 μL of anhydrous ethanol, thereby generating a 5000× concentrate (i.e., 5000 times more concentrated than the original water sample). Optima LC/MS water (Thermo Fisher Scientific, San Jose, CA) was used as an extraction blank for quality control. Based on the previous works of Morandi et al.,^18,33^ the total organic carbon of BML OSPW can range from 41.5–150 mg/L, and we can estimate that the recovery of acidic and neutral compounds by the current method is 82%.

### Analysis by HPLC-HRMS

Chromatographic separation was achieved with an HPLC Accela System (Thermo Fisher Scientific, San Jose, CA) and a C18 Gold column (100 × 2.1 mm, 1.9 μm particle size, Thermo Fisher Scientific, San Jose, CA) at 40 °C. The flow rate was 0.5 mL/min, and the injection volume was 3 μL in both ionization modes. Each extract was diluted to a 50× concentrate in 100% methanol for injection. The mobile phases were (A) 0.1% acetic acid in water and (B) 100% methanol. The elution gradient started with 5% B and 95% A for 1 min, then a linear ramp increasing the proportion of B to 90% at 9 min and to 99% B over 5 min, and finally returning to 5% B in 1 min and equilibration for 4 min.^34^

The HRMS was an Orbitrap Elite (Thermo Fisher Scientific, San Jose, CA) operating in electrospray ionization mode and set to a nominal resolving power of 240,000 at *m/z* 400. For qualitative profiling of the complex organics in all water samples, two separate injections of each sample were made to allow detection of organic acids in negative ionization mode and polar organic neutrals and organic bases in positive ionization mode. Ionization potential was set at ±4 kV, with sheath, aux, and sweep gas flows at 40, 25, and 2 (arbitrary units), respectively. Vaporizer and capillary temperatures were at 350 and 325 °C, respectively. Based on previous studies involving the comprehensive analysis of OSPW, the mass range was set from *m*/*z* 100–500 in negative mode and from *m/z* 160–500 in positive mode.

To characterize the extractable organics of water samples, features were assigned chemical formulas using Xcalibur software (Thermo Fisher Scientific, San Jose, CA) for the retention windows of 7–13 min for negative mode and 3–13 min for positive mode. Kendrick Mass and Kendrick Mass defect values, normalized to CH_2_, were calculated from accurate *m/z* measurements, and the response of each was corrected for the response of the same features in blanks (i.e., LC/MS water extract) when necessary. If there was a corresponding signal in blanks, a signal greater than 3× the blank was required to be accepted as a true signal in the sample. Mass tolerance for formula assignment to each detected chemical species was set to ±5 ppm, and the average ppm error was 1.63% across environmental samples (Table S1). Species were binned into the heteroatomic formula class in each ionization mode (e.g., O_2_^–^, O_2_^+^, SO_2_^–^, NO^+^) for broad chemical profiling.

### Cytotoxicity

The human hepatocellular carcinoma cell line, HepG2 [HEPG2] (American Type Culture Collection HB8065, Manassas, VA) was grown in Eagles Minimum Essential Media (30–2003, American Type Culture Collection, Manassas, VA) supplemented with 10% fetal bovine serum (Sigma-Aldrich, Oakville, ON, Canada) and 1% penicillin–streptomycin (100 U/100 μg/mL, Invitrogen, Carlsbad, CA). Cell culture details are in the SI.

Cytotoxicity was measured using real-time cell analysis (RTCA) (ACEA Biosciences, San Diego, CA), a label-free, continuous, long-term cytotoxicity assay using viable cells that have some advantages over colorimetric assays.^35,36^ Established RTCA methods are more common for pharmaceuticals,^37,38^ though programs, such as the National Toxicology Program and the US EPA, have implemented RTCA in their regimen to assess cytotoxicity efficiently for many environmental chemicals.^36,39^ Experimental details and theory of the technique are described in the SI, though briefly, adherent cells in each well correlate with impedance, and this signal is converted to the unitless parameter, cell index (CI), thereby representing an integrated measure of cell growth, morphology, and adhesion. Four replicate wells per plate were used for each dose, and each environmental sample was tested in triplicate (i.e., using three plates). Measurements were taken every hour over a period of 100 h in the incubator, generating a dynamic response profile which was later analyzed with GraphPad Prism 7. A solvent control of 0.25% (v/v) anhydrous ethanol was used, matching the highest extract dose.

### Endocrine Activity

The commercial XenoScreen XL YES/YAS kit (Xenometrix, Allschwil, Switzerland) was used as received. Genetically modified Baker’s yeast (*Saccharomyces cerevisiae*) has the human estrogen receptor (hERα) or androgen receptor (hAR) integrated into the main chromosome and a plasmid with the lacZ reporter gene, encoding β-galactosidase as well as an estrogen (YES) or androgen (YAS) response element.^40^ Experimental details are in the SI, but briefly, YES and YAS strains were grown from frozen in vented T25 flasks for 48 h in an orbital shaker at 100 rpm in a 31 °C incubator.^40^ To prepare positive control hormone solutions, 100 μL of anhydrous ethanol was used to dissolve 17β-estradiol (E2), 4-hydroxytamoxifen (4-HT), 5α-dihydrotestosterone (DHT), and flutamide (FL). The yeast was incubated with the treatments for 18 h, with color development occurring following another 1 h of incubation. Doses were tested in duplicate.

The Varioskan LUX plate reader (Thermo Fisher Scientific, San Jose, CA) was used in absorbance mode with optical densities (OD) measured at wavelengths of 570 nm (OD_570_) and 690 nm (OD_690_). OD_690_ measures yeast growth and also functions as a correctional value for diffraction when OD_570_ is measured for color development. A solvent control of 0.67% (v/v) anhydrous ethanol was used, matching the highest extract dose. Agonists are defined as an increase from negative control, while antagonists are defined as a reduction from the agonist baseline, whereby a known and equal amount of E2 or DHT is in all wells, allowing inhibition from the agonist baseline to be observable. Reduction ratios (*R*_R_) were calculated from the agonist baseline control as described in the SI.

### Statistical Analyses

To quantify the RTCA response profiles, inhibitory concentration (IC_50_) histograms were plotted over time.^41^ To generate the IC_50_ histograms, two different concentration scales were used to describe the dose of organics. First, an enrichment factor scale (×) was used to express the concentration of organics relative to environmental concentrations in the original water samples. For example, a 1× dose is equivalent to the original sample, while 10× is 10-fold higher. Second, to enable comparing the toxic potency among samples, we also expressed concentration on the basis of the dried mass of organic extract (i.e., mg organics/L). Thus, concentrations (either units of ×, or mg of organics/L) were log-transformed and normalized from 0 to 100%, where 100% was the normalized cell index of the negative control at every time point measured. Next, a nonlinear fit with the formula of “log (inhibitor) versus normalized response (variable slope)” was used, and standard error of the mean (SEM) was calculated. The generated IC_50_ values at each time point were plotted on an IC_50_ vs time (h) plot for a total of three replicate plates to calculate a mean and SEM. A two-way ANOVA was used, grouped into OSPW sample and time as factors, with a post-hoc Tukey test for multiple comparisons (α = 0.05).

To quantify the endocrine activity, the mg/L concentration of the sample extracts and positive controls were log-transformed. In preliminary experiments, no agonist activity was found (data not shown); therefore, only the methodology and results for antagonist activity were included here. Reduction ratio values were normalized with 0% set as the highest dose of the antagonist hormone, flutamide or 4-hydroxytamoxifen, when the greatest degree of antagonism occurred, while 100% was the lowest dose of antagonist hormone that had negligible antagonism. Next, a nonlinear fit with the formula of “log(inhibition) versus response” was used. An equivalence ratio (EQ) was calculated from the EC_50_ of the sample and the EC_50_ of the hormonal positive control to assess toxic potency (SI).

## Results and Discussion

### Chemical Characterization of Extractable Organics

**Figure 1:**
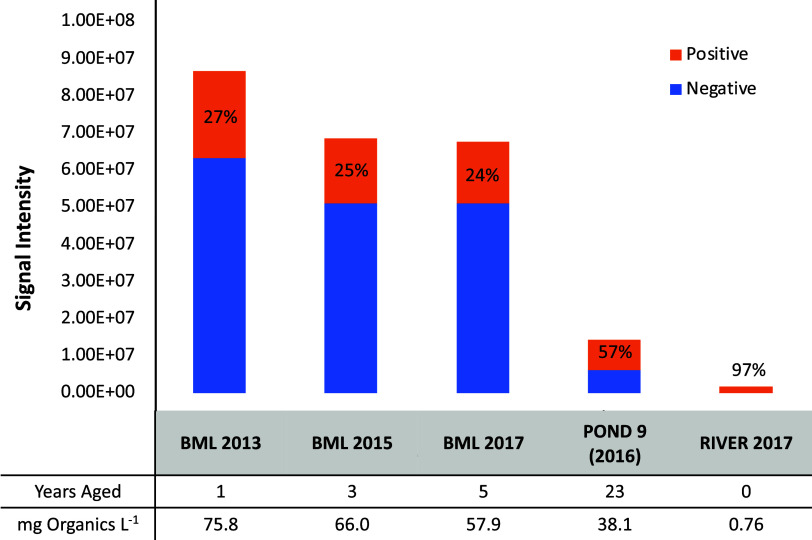
HPLC-HRMS total ion intensity in negative and positive ion modes (*n* = 1) of environmental samples and the corresponding organic extract concentrations (mg organics/L) and years aged. The proportion of positive ion mode species to the total signal intensity is indicated by percentages.

BML 2013 had the highest gravimetrically determined concentration of extracted organics among all samples examined (75.8 mg/L), approximately 100-fold higher than for the reference sample from the Athabasca River (Figure 1). BML samples collected in later years and the field-aged experimental Pond 9 had lower extractable organic concentrations that generally declined with the number of years of aging in the field (Figure 1).

**Figure 2:**
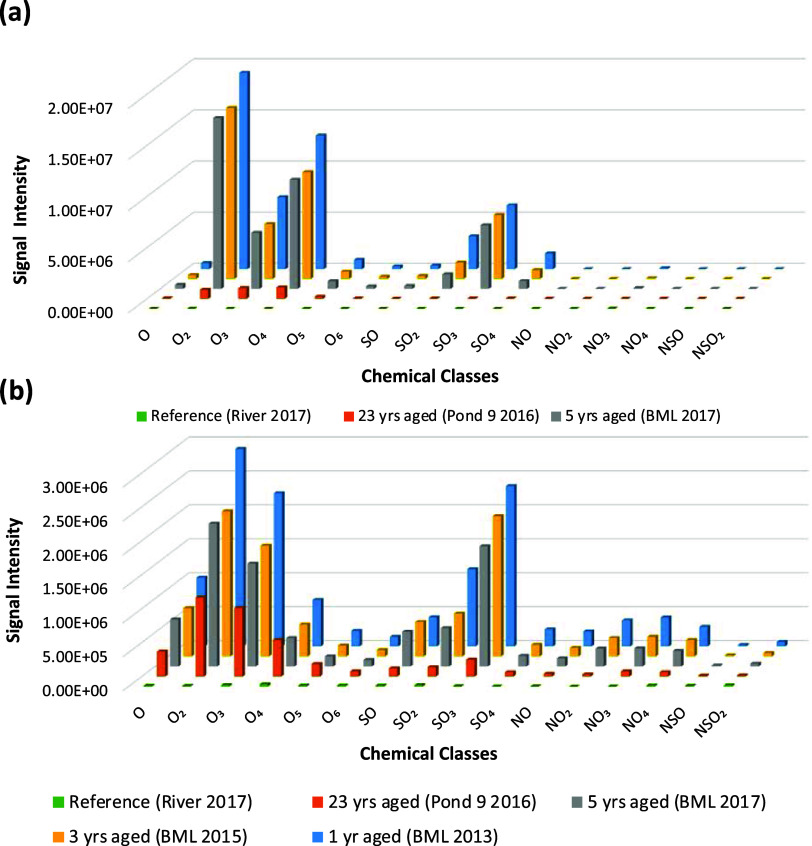
Heteroatomic chemical class distribution of different environmental samples in (a) negative and (b) positive ionization modes. The signal intensities are absolute values for each chemical class in each sample (*n* = 1).

Consistent with gravimetric concentrations of organics, injections of BML 2013 to HPLC-HRMS produced the highest total ion intensities in both positive and negative ionization modes (Figure 2), and the intensity decreased with aged BML samples and in Pond 9. Importantly, the total ion intensity in negative mode decreased to a greater extent in aged samples than the corresponding signals in positive mode. Thus, the relative proportion of positive mode species increased from 27% in BML 2013, 25% in BML 2015, and 24% in BML 2017 to 57% in Pond 9 (Figure 2). This suggested that the polar OSPW species detected in positive ionization mode may be more persistent than organic acid species detected in negative mode, including NAs. As expected, HPLC-HRMS total ion intensity for the reference Athabasca River sample was much lower than any of the bitumen-impacted samples in both ionization modes. The Athabasca River reference sample was to test representative freshwater that was not (i.e., could not physically have been) impacted by seepage of OSPW. It is known from our past work and previous public reports that OSPW seepage has impacted McLean Creek.^31,32^ Thus, the current reference sample had to be taken upstream of McLean Creek, and A20e SW was close to this.

Among all BML samples analyzed in negative ion mode, there were similar proportions of O_2_^–^ > O_4_^–^ > O_3_^–^ (Figure 2a). This is consistent with simple dilution being responsible for the decreasing absolute intensity of the total signal with aging. In Pond 9, chemical class distribution was different than BML, whereby the O_4_^–^ class was most prominent, followed by O_3_^–^ > O_2_^–^, and the overall intensity was lower than in any BML sample (Figure 2a). This contrasting chemical signature likely results from *in situ* degradation processes active in this small-scale demonstration pond over its 23 years of aging. Compared to BML, this experimental pond is relatively shallow; thus, photodegradation may have a greater impact here than in deeper end-pit lakes.^42^ Pond 9 is also different from BML because it was initially composed only of OSPW, without underlying fine tailings; thus, the water is less turbid, and any degradation of the organics is rather simple to detect due to no upwelling of contaminated pore water. BML contains a deep layer of fine tailings capped with a surface layer OSPW. The underlying fine tailings are known to slowly compress over time and in so doing, release fresh OSPW-like pore water into the surface layer,^20^ which might mask the effect of any slow degradation processes at the surface. OSPW samples have spatial and temporal heterogeneity between tailings pond sites and even within individual tailing ponds.^43^ Therefore, the difference in OSPW sources between BML and Pond 9 could contribute to differences in chemical composition and toxicity, though these are the only available samples to test hypotheses on *in situ* aging of authentic OSPW.

In the negative ionization mode, there were only minimal differences between the absolute intensities and relative chemical class profiles for BML 2015 and 2017, but the general trend compared to BML 2013 was a decrease in ion intensities over time (Figure 1). In positive ion mode, there were more differences in absolute intensities between BML 2015 and 2017, particularly for the SO_3_^+^ and O_3_^+^ classes (Figure 2b). For all OSPW samples, there was a wider contribution of chemical classes in positive mode to the overall intensity, i.e., O*_x_*^+^, SO*_x_*^+^, and NO*_x_*^+^, though the general trend was again a decrease in intensity for OSPW that had been aged longer in the field. However, while the total intensity decreased with age, the relative proportion of chemical classes in all OSPW samples in positive mode, including the aged Pond 9, did not greatly change. This is different from Pond 9, analyzed in negative mode, which had unique relative proportions of chemical classes compared to BML, likely indicative of *in situ* degradation processes of the organic acids. Therefore, non-acid polar species detected in positive mode may be more recalcitrant, with declining absolute intensities therefore being suggestive of dilution. Heteroatomic chemical class distributions grouped by the sample are shown in Figure S2.

### Cytotoxicity of BML 2013–2017

The blank quality control extract showed no cytotoxic effects on HepG2 cells (Figure S4b), and neither did the extract of water from the Athabasca River, even at doses up to 12.5× (Figure S4a). In contrast, Figure S3 shows the RTCA response profile of HepG2 cells treated with various BML and Pond 9 extracts, demonstrating that the bitumen-derived organics in OSPW are cytotoxic to HepG2 cells. This basic finding is notable, given very little available human or mammalian toxicity data for OSPW to date and novel use of the RTCA system.

**Figure 3:**
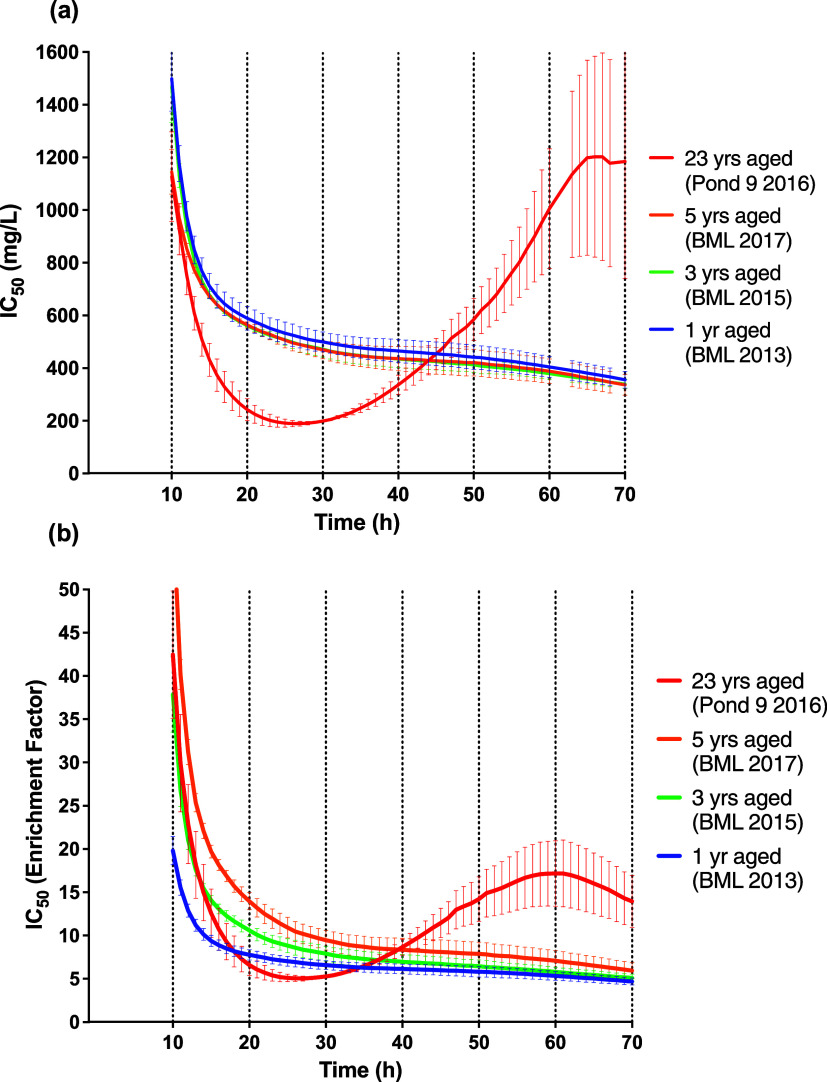
Temporal IC_50_ histograms for HepG2 cells exposed to aged BML and OSPW water sample extracts in the RTCA assay, with the data normalized on two different y-axes scales: (a) mg extract/L and (b) enrichment factor (×). Data are plotted as mean ± SEM for triplicate plates. Some IC_50_ values for Pond 9 on the mg/L scale were not able to be calculated by GraphPad after 60 h; thus, all data at those times were excluded from the plots for ANOVA calculations.

BML extracts all demonstrated a decrease of the IC_50_ with RTCA assay time duration (Figure 3). The relative cytotoxicity between samples was BML 2013 > BML 2015 > BML 2017, whereby all BML samples had significantly different IC_50_ values from each other at 10 to 14 h (Tables S3 and S4). Therefore, for the three BML samples, cytotoxicity decreased with increased aging, which is reasonable, given the lower organic concentrations (yet similar chemical profiles) in aged samples (Figure 2), as discussed above. This result is also consistent with Syncrude Canada Ltd.’s reported acute toxicity testing of BML water to fathead minnow and rainbow trout between 2013–2016,^44^ wherein adverse effects were observed in 2014 but not thereafter, up to and including 2018.^19^

Overall, the acute IC_50_ values for BML samples ranged from 6× to 10×, well above concentrations found in the field. While this enrichment factor scale is an effective means of evaluating environmental relevance (i.e., 1× is the concentration in the field), it is not useful for benchmarking the toxic potency among samples. Therefore, to compare toxic potency, we report the same doses as the mass of extract per unit volume (i.e., mg organics/L) based on gravimetric concentrations (Figure 1). After this normalization, the declining toxicity described above between BML samples was no longer significant, other than between BML 2013 vs 2017 at 10 h only (Tables S5 and S6). In other words, the toxic potency of the dissolved organics extracted from BML samples was not different, suggesting that simple dilution of the organics can account for cytotoxicity differences among BML samples of different ages over the first 5 years. As discussed above, this is supported by the high similarity in chemical class distributions among BML samples.

With the finding that the SEM of the 1× dose of BML organics overlapped with negative control (Figure S3), a threshold measure was made based on an IC_10_ analysis at each time point (Figure S5). Here, the IC_10_’s approached, but were not below, the respective field concentrations of BML 2013 (75.8 mg/L), BML 2015 (66.0 mg/L), and BML 2017 (57.9 mg/L). This indicates a lack of cytotoxicity for the organics at the field-relevant dose of 1× and that only above this concentration would cytotoxicity become observable.

Dompierre et al.^45^ modeled the change in water volume and chemical mass balance for BML between 2013 and 2015. While the water balance was rather constant over time, slight improvements to overall water quality were attributed to dilution, which was described as 5–10% dilution per year. This estimated rate of dilution is consistent with changes reported here in the gravimetric concentrations of extracted organics between BML 2013 and 2017, with, on average, a 6% reduction in organic mass per year (Figure 1). More explicitly, Dompierre et al.^45^ described that the largest water input to BML was fresh water from a local reservoir, which served to dilute the lake, and that the water balance in BML was maintained by pumping excess OSPW at the surface into the industrial bitumen extraction process,^45^ thereby representing an unnatural removal mechanism for the end-pit lake OSPW.

### Cytotoxicity of Pond 9

On an enrichment factor scale, Pond 9 displayed an interesting biphasic IC_50_ profile (Figure 3). More specifically, over the first 24 h, the extract of this aged water appeared more toxic than lesser-aged BML extracts, with Pond 9 IC_50_’s showing significantly more toxicity than BML 2017 (from 10 to 26 h) or BML 2013 (from 10 to 13 h), and not significantly different from BML 2015 (Tables S3 and S4). However, by 46 h, the Pond 9 extract showed significantly less toxicity than all BML extracts for the remainder of the assay duration. The subsequent decrease in IC_50_’s for Pond 9 after 60 hrs (Figure 3) should not be overinterpreted; this was likely due to a lack of nutrients remaining in the culture media or cell senescence. The toxicity profile of the Pond 9 extract maintained this biphasic profile after converting it to a potency scale (mg/L). At this level of comparison, Pond 9 cytotoxicity was not significantly different from any BML extract from 11 to 16 h, suggesting the same toxic potency in this time frame despite different ages, with Pond 9 starting to have a significantly higher toxic potency than BML samples at 17 to 30 h (Tables S5 and S6).

Although Pond 9 had a lower concentration of NAs and extractable dissolved organics compared to BML, the organics that remained in Pond 9 after 23 years of aging were more potent at inducing toxicity during the initial phases of the exposure. This highlights the importance of comprehensively monitoring the chemical composition of aging OSPW in end-pit lakes. Monitoring of NA concentrations alone has long been the industry and government standard for OSPW remediation but will unlikely be predictive of water toxicity in real-world end-pit lakes containing OSPW and fine tailings. Considering heteroatomic class distributions (Figure 2), Pond 9 had a unique distribution of chemical classes compared to BML, characterized by very low NA concentrations in negative mode, and in positive mode, the O^+^, O_2_^+^, O_3_^+^, and O_4_^+^ classes made a much greater relative proportion to the remaining total mixture. These non-acid species may exert a unique mode of action that is masked by antagonism (i.e., due to high concentrations of other substances) in fresh OSPW samples, or these may be bioactivated degradation products from other organics, such as from residual bitumen in the aquatic systems.

The biphasic cytotoxicity response for Pond 9 (Figure 3) may be due to transient narcosis. Narcosis is the suspected mechanism of action for total OSPW organics, and with Pond 9 having a lower concentration of these organics, the biphasic effect may represent recovery, as narcosis is known to be reversible.^46^ However, the exposure is not removed in the assay here and therefore does not give the opportunity for recovery to occur. Previous RTCA studies have grouped chemicals by mechanisms of action based on their temporal response curves,^37,38,47^ and a similar biphasic profile has been shown for an antimitotic subgroup of chemicals, including the drug monastrol.^37^ Cell cycle arrest at mitosis is characterized by cell rounding, which can lead to transient detachment of the cells. For cell lines that lack a robust mitotic checkpoint, such as the cancer cell line HepG2, “mitotic slippage” can occur, whereby a subpopulation of cells escape the initial arrest, resulting in a subsequent recovery of cell growth.^37^ Xi et al.^38^ clustered 47 chemicals into similar mechanisms of action to HepG2 cells based on their RTCA profiles and used Vinblastine sulfate (a chemotherapy drug) as a representative compound for a biphasic response, classifying it as targeting tubulin, which is similar to monastrol.^37^ When combining RTCA with a micronucleus flow assay, Vinblastine was shown to be an aneugen, resulting in daughter cells with an abnormal number of chromosomes. Therefore, further investigation of the mechanism of action of Pond 9 extract may be warranted to clarify its toxicological similarities to vinblastine and monastrol and not solely assume transient narcosis.

Based on previous work, there is generally declining toxicity to aquatic species as OSPW ages, but some persistent toxicity remains in the aged samples.^3,15−17,48^ There are some exceptions, where studies have shown no difference between fresh and aged OSPW. For example, Marentette et al.^49^ tested NA extracts from fresh and aged OSPW for their effects in fathead minnow embryos and reported similar toxicities, even though the aged sample had a relatively low proportion of NAs. Bartlett et al.^50^ later performed a number of bioassays with fresh and aged OSPW and also reported an aged sample to be similarly toxic, or more toxic, than fresh samples for most aquatic species. The consistent result of these studies is that *in situ* aging is not an effective OSPW remediation strategy on its own, even when considered on a time frame of >20 years.

### Endocrine Activity

**Figure 4:**
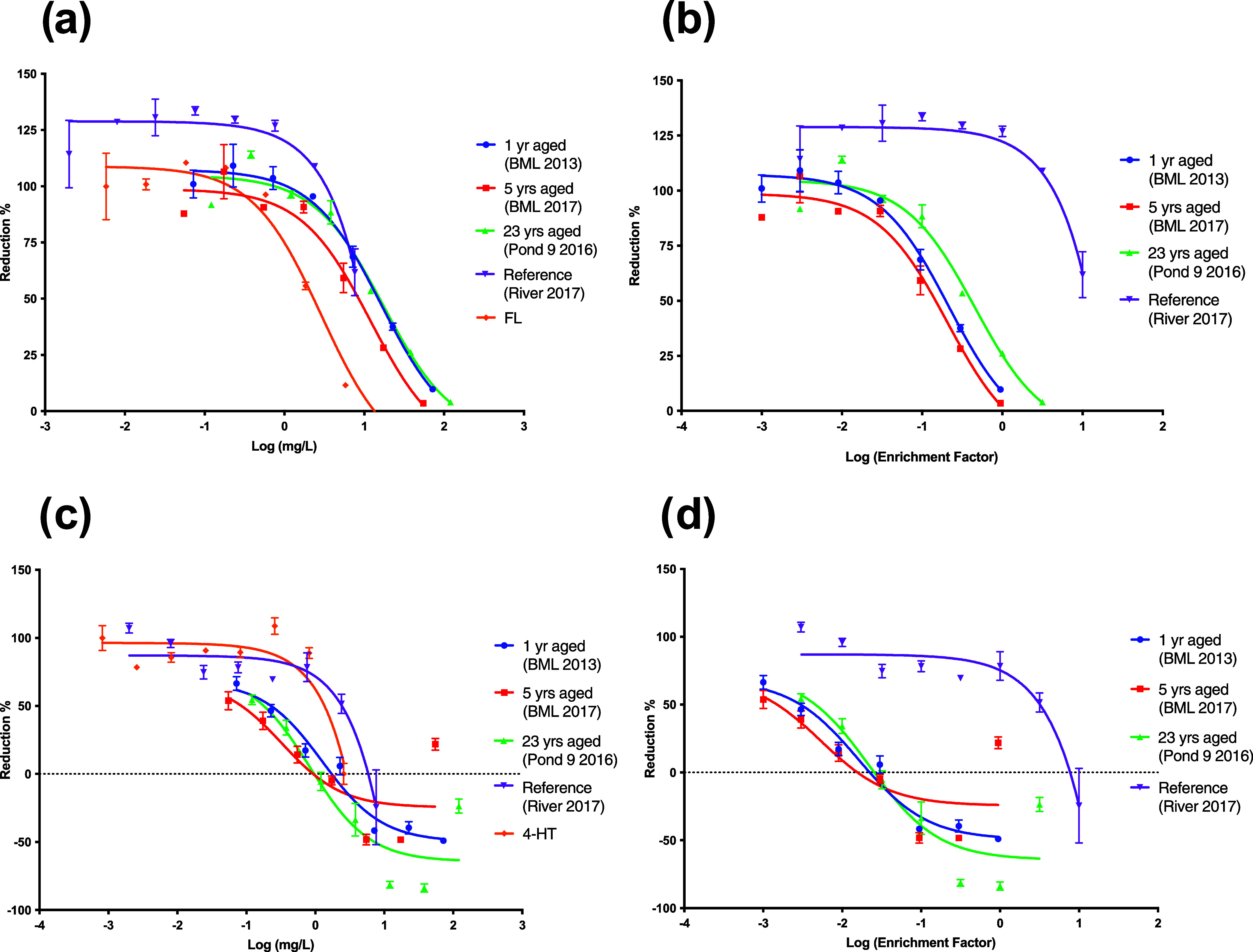
YAS antagonism on (a) a mg/L scale and (b) enrichment factor (×) scale, as well as YES antagonism on (c) a mg/L scale and (d) enrichment factor scale. Reduction % is normalized to the antagonistic positive control for the assay, where 0% is the highest dose and 100% is the lowest dose. Only on the mg/L scale can the extracts be compared to the positive control hormone of 4-hydroxytamoxifen (4-HT) for YES antagonism and flutamide (FL) for YAS antagonism. Values are the mean ± SEM (*n* = 2).

In preliminary work, BML samples contained no ER or AR agonist activity (data not shown), consistent with Leclair et al.’s^21^ testing of another experimental pond at Syncrude Canada, Pond 10. Thus, agonism is not discussed further; rather we focus on ER and AR antagonist activity, which was present in all OSPW extracts. Dose–response curves are shown in Figure 4, plotted by enrichment factor (×) for evaluating environmental relevance, and on a mg extract/L scale to compare potency and to benchmark the effect against positive controls. Dose selections for testing of endocrine activity were based on observed sublethal thresholds in preliminary work (Figure S6); thus, BML doses did not exceed 1×, Pond 9 doses did not exceed 3×, and Athabasca River doses did not exceed 10×. In both YES and YAS assays, all OSPW-related samples produced an antagonistic response at doses that were 2–3 orders of magnitude lower than required to elicit a response with the reference extract, River 2017 (Figures 4b,d). In general, on both enrichment factor and concentration scales, there were slight differences in the YES and YAS antagonistic effect between OSPW aged 1 year (BML 2013), 5 years (BML 2017), or 23 years (Pond 9). BML 2015 was not tested in the YES/YAS assay due to a lack of space in the multiwell plates.

Quantitative EC_50_ calculations allowed for ranking of potency (mg extract/L), with YES antagonism resulting in the highest potency for the most aged BML sample (BML 2017), followed by Pond 9 > BML 2013 > River 2017 (Figure 4, Table S2). YAS antagonism resulted in similar results and ranking, except that Pond 9 and BML 2013 had similar potencies.

For the River 2017 extract, the YES EC_50_ was extremely high (167× above field concentration), while its YAS EC_50_ could not even be accurately calculated due to low effects (EC_50_ estimated to be >40,000×), demonstrating negligible endocrine activity and clear differences compared to OSPW-related samples which had effects at environmentally relevant doses. The EC_50_’s for ER and AR antagonism of BML 2013, BML 2017, and Pond 9 were all below their field concentrations (Figure 4, Table S2). Although there was minor variability between the BML and Pond 9 samples, the EC_50_’s overall were quite similar despite differences in aging and differences in the chemical profile for Pond 9 (Figure 2).

The EC_50_ values for positive control hormones were used as a benchmark to determine equivalence (EQ) and were either experimentally determined or theoretical, based on commonly fitted values from Xenometrix’s workbook (SI). For YES, BML 2017 and Pond 9 2016 were both more potent on a mg/L scale than the positive control 4-hydroxytamoxifen, indicated by EQ values below 1, while BML 2013 had an EQ of 1.03 (Table S2). For YAS antagonism, none of the extracts were below 1 for flutamide EQ.

Considering no change in ER/AR antagonism with aging, and the increased relative proportion of chemical species observed in positive ionization mode, we speculate that the ER and AR antagonism of Pond 9 is largely driven by the polar non-acid species, including O^+^, O_2_^+^, O_3_^+^, and O_4_^+^ classes. There may be mixture antagonism in the whole (unaged) OSPW organic mixture, and selective degradation of the acid species may lead to enhanced endocrine activity of the non-acid chemicals in aged waters, such as Pond 9. It is also important to note that many of the other SO*_x_*^+^ and NO*_x_*^+^ chemical classes remaining in Pond 9 may also contribute to the endocrine activity.

Compared to the literature, Leclair et al.^21^ tested 17-year field-aged OSPW from Pond 10 with YES/YAS and only detected antiestrogenic and antiandrogenic effects; however, their chemical isolation method was specific for NAs, and the extract was only characterized by mass spectrometry in negative ionization mode. They also generated four fractions with different NA content, and fractions with a higher aliphatic NA content appeared most potent, with all fractions generating an EC_50_ ranging from 1.5–16.4 mg NAs/L for YAS antagonism and 0.5–9.6 for YES antagonism. In the current investigation, Pond 9 extract generated an EC_50_ of 17.2 mg/L for YAS antagonism and 0.83 mg/L for YAS antagonism. These results are consistent with Leclair et al.^21^ in that YES antagonism is more potent than YAS antagonism and that the determined EC_50_ values are in a similar range, although their fractionated samples may be more potent due to isolation of the antagonists from other OSPW chemical species.

The persistent YES and YAS antagonism with OSPW aging is a warning against relying only on *in situ* degradation processes for the end-pit lake strategy. Even though the cytotoxic IC_50_’s calculated for the BML organics were above field concentrations in the current work (thresholds marginally above 1×), the sublethal endocrine active effects had measurable potencies far below field concentrations in these *in vitro* tests, no matter the age of the OSPW sample. This is surprising considering that Pond 9 has been aged 23 years and is a best-case scenario for what BML water chemistry could look like in the future, yet this water was equally as endocrine active as contemporary samples of BML.

Nevertheless, it is important to distinguish between mechanistic potency generated from *in vitro* tests based on disturbances to molecular pathways and potency for an adverse effect in a living organism. The US EPA^51^ in 2010 released a guidance document for evaluating endocrine disruptors and argued that a hypothesis-driven weight of evidence framework is necessary to examine and integrate studies from different biological levels to determine endocrine activity.^52^ Defining the adverse effect potency in living organisms is beyond the scope of this research, but what we can conclude is that OSPW extracts here had endocrine activity that was as potent as positive control drugs, even for aged samples. This warrants further testing and evaluation to contribute toward a weight-of-evidence approach to assess the effectiveness of aging as a remediation strategy for removing endocrine activity in OSPW.

## Conclusions

Dilution is effective in reducing the toxicity of any environmental contaminants, and it is often used to reduce the concentrations of a contaminant to below the threshold of a toxic effect. However, total OSPW stored by today’s oil sands industry is estimated to account for 0.5% of the volume of downstream Lake Athabasca,^53^ Canada’s 8th largest lake, so dilution of this amount of OSPW may not be an acceptable strategy at a time when water withdrawals already put high pressure on the Athabasca River, especially considering growing stress from climate change and a dwindling Athabasca glacier.^54^ Also, the rationale and goal of the end-pit lake strategy were not originally to allow for dilution but rather to allow for sedimentation of fine tailings and degradation of OSPW organics over time.^13^ Continuous dilution and coagulant additions to BML and 30 other planned end-pit lakes could represent an unacceptable and expensive reclamation strategy that negatively impacts future generations of Canadians. New strategies or additional treatment of OSPW within end-pit lakes are likely necessary to achieve the ultimate goal of a biologically productive lake connected to the natural watershed.^13^

## Supporting Information Available

A map of the reference location (Figure S1) and details of the materials and methods for sample collection, cell culture, RTCA, and YES/YAS. Foundational results include the heteroatomic chemical class distributions of each sample in positive and negative mode (Figure S2), the average ppm error for HRMS analysis (Table S1), RTCA toxicity response profiles for environmental samples (Figure S3) and controls (Figure S4), RTCA IC_10_ threshold measurements (Figure S5), yeast growth (Figure S6), endocrine activity quantification (Table S2), and RTCA ANOVA results (Table S3–S6) (PDF)
