# Supplementary material for: Persistent Cytotoxicity and Endocrine Activity in the First Oil Sands End-Pit Lake
Source: ACS ES T Water. 2023 Jan 24;3(2):366–76. doi: 10.1021/acsestwater.2c00430 (PMC11181316; doi:10.1021/acsestwater.2c00430)
Supplement: Supplementary file 1 — ew2c00430_si_001.pdf [file ew2c00430_si_001.pdf]

Persistent Cytotoxicity and Endocrine Activity in the First Oil Sands End-Pit Lake

Ian G.M. Gault[a], Chenxing Sun[a,1], Jonathan W. Martin[b,*]

[a] Division of Analytical and Environmental Toxicology, University of Alberta, Edmonton, AB, T6G 2G3, Canada
[b] Department of Environmental Science, Stockholm University, Stockholm, 106 91, Sweden

* To whom correspondence should be addressed:
Jonathan W Martin, jon.martin@aces.su.se, phone: +46721462773

[1] Current address: Environmental Monitoring and Science Division, Alberta Environment and Parks. Edmonton, AB, T5J 5C6, Canada.

Supporting Information

42 pages, 6 figures, 6 tables

MATERIALS AND METHODS

Sample collection methods. Surface OSPW samples were collected with a Van Dorn water sampler from an outflow barge between 0.5 and 1 m below the surface. The river water sample was collected approximately 30 cm below the surface in an acid-cleaned 2.5 L polypropylene jug, with the cap removed and secured below the surface to avoid contamination by the surface microlayer.

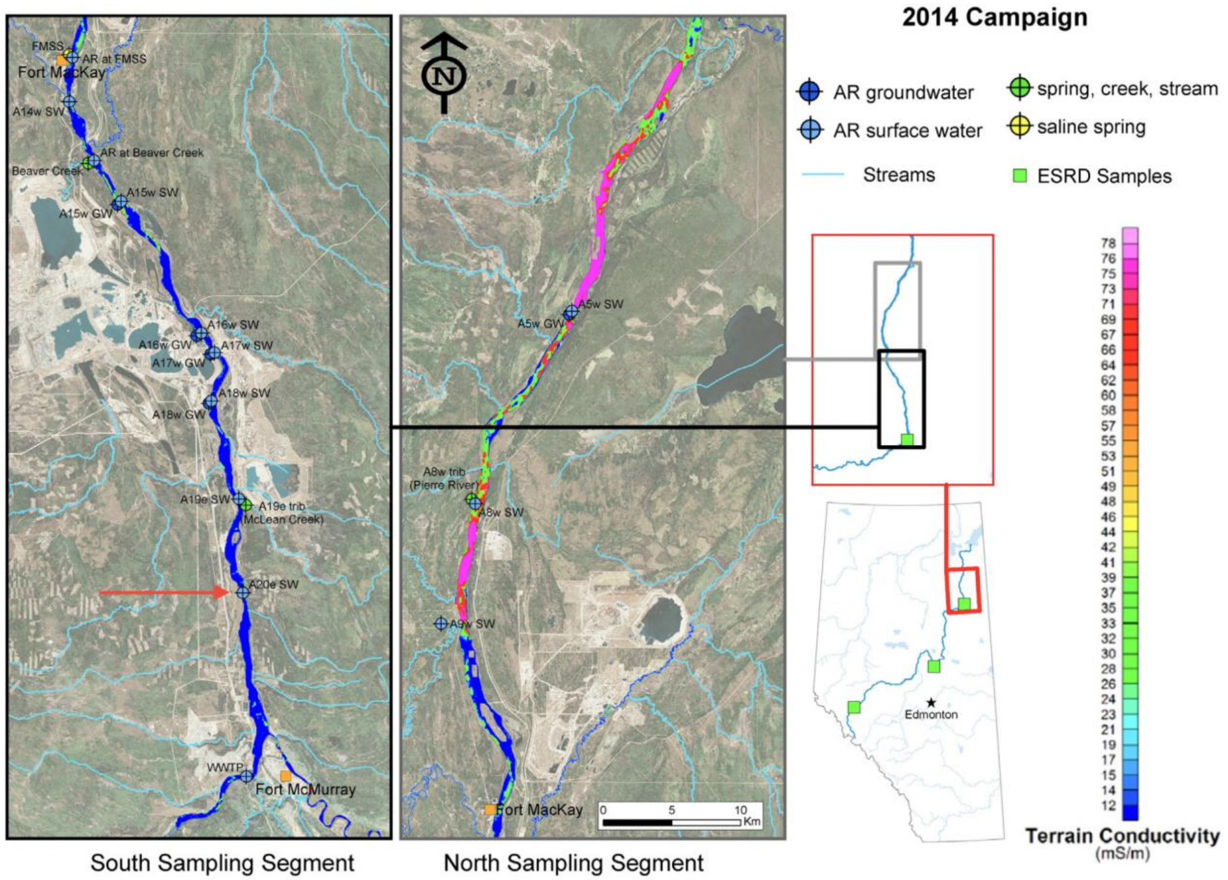

Figure S1: Sample collection location of the Athabasca River water sample, A20e SW (highlighted by red arrow), which is upstream of the oil sands industry. Figure reprinted from Science of the Total Environment, 580, Shotyk W, Bicalho B, Cuss CW, et al.,[1] "Trace metals in the dissolved fraction (< 0.45 µm) of the lower Athabasca River: Analytical challenges and environmental implications," 660-669, Copyright (2017), with permission from Elsevier.

Cell culture method. HepG2 cells were maintained at 37°C in 5% CO_2 and sub-cultured twice weekly into standard 10 cm x 20 mm cell culture dishes (Corning Inc., Corning, NY). Dulbecco's phosphate buffered saline (Thermo Fisher Scientific, Burlington, ON, Canada) was used to wash the cells, and 0.25% Trypsin-Ethylenediaminetetraacetic acid (Thermo Fisher Scientific, Burlington, ON, Canada) was used to detach the adherent cells from cell culture dishes.

Real Time Cell Analysis (RTCA) method. Our labs have previously assessed nanoparticles[2] and water disinfection byproducts by RTCA,[3] but to the best of our knowledge RTCA has not previously been applied to OSPW samples until now. RTCA consists of a 96-well plate with microelectrodes covering the bottom surface of each well. A current is passed through the wells at three different frequencies: 10 kHz, 25 kHz, and 50 kHz, measuring changes in impedance (Z) over time at the electrode-media interface of each well.[2] Three frequencies allows for the maximization of Z, which is converted to resistance (R) by the system analyzer through the following equation:

$$Z = R + jX$$

Here, j is the imaginary component and X is the reactance. R is then converted to the unit-less parameter called Cell Index (CI) through the following equation:

$$CI = \max_{i=1,\ldots,N}\left(\frac{R_{cell}(f_i)}{R_b(f)} - 1\right)$$

With $R_{cell}(f_i)$ as the frequency-dependent resistance when cells are attached to the microelectrode, $R_b(f)$ as a reference value for when cells are no bound, and N as the number of frequencies that Z is measured under. This means that as the $R_{cell}(f_i)$ value changes over time due to cell attachment and proliferation, so does CI.[2] Therefore, CI is a representation of cell-electrode contact. Cells are seeded and allowed to proliferate in clean wells for ~24 h, at which point the CI is normalized, i.e., NCI, to reduce inter-well cell number variability.

For HepG2 cells, an optimized seeding density was determined to be 12,500 cells/well (data not shown). Once a CI of 1 was achieved, the CI values were normalized to reduce inter-well cell number variation, and this was termed the normalized cell index (NCI). The NCI allowed differences in cell growth to be attributed to the various treatments, and not to slight variations in initial cell number between wells. The NCI values collected hourly over the exposure period were plotted automatically through the RTCA system analyzer to create time-dependent cellular toxicity response profiles. Treatment effects causing apoptosis, necrosis, morphological changes, or uncontrolled proliferation can be reflected in the toxicity response profile.[2]

Each treatment has 4 replicate wells, which are averaged for comparison to other treatments or controls within the plate. Thus, every averaged toxicity response profile has a standard error of the mean (SEM). Measurements are taken every hour over a period of 100 h while cells are still in the incubator, generating a dynamic response profile which is visualized and analyzed with GraphPad Prism 7. Negative control wells contained media only, allowing for maximal cell growth, while the solvent control included 0.25% (v/v) anhydrous ethanol. Arsenic (III) at a concentration of 250 μM was used as a positive control, as it is a well-studied cytotoxic chemical that leads to cell death resulting in a NCI of 0.[4] It is therefore a useful benchmark for comparison. The positive control was prepared from sodium arsenite (Sigma-Aldrich, Oakville, ON, Canada) in deionized water.

A dilution scheme of anhydrous ethanol was first tested to find an appropriate concentration range of ethanol in media (% v/v) that is sufficient to dissolve the organics but not cause any cytotoxic effects. The optimized protocol used 200 μL of anhydrous ethanol, resulting in 0.25% ethanol (v/v) at the highest treatment dose (12.5×) from which further dilutions were created, maintaining the ratio of organic extract to solvent constant throughout all dilutions.

Yeast Estrogenic and Androgenic Screen (YES/YAS) Method. Genetically modified Baker's yeast (*Saccharomyces cerevisiae*) have the human estrogen receptor (hERα) or androgen receptor (hAR) integrated into the main chromosome and a plasmid with the lacZ reporter gene, encoding β-galactosidase as well as an estrogen (YES) or androgen (YAS) response element.[5] Once a ligand is bound to its receptor, the complex can bind to its respective response element on the plasmid, allowing for expression of the β-galactosidase enzyme. From here, the use of lyticase and a detergent, termed the lacZ reaction mixture, allows for secretion of the enzyme into the extracellular media, catalyzing the reaction of the yellow substrate, chlorophenol red-β-D-galactopyranoside (CPRG), to its red product. This is quantified with a plate reader as an indicator of the degree of hERα or hAR agonism or antagonism.

For the YES/YAS assays, the percentage of anhydrous ethanol in the positive controls was set by the supplier at 0.67% (v/v). Thus, the highest treatment dose (10×) was also prepared with a final concentration of 0.67% (v/v) ethanol, while maintaining a consistent ratio of organic extract or control hormone to solvent in each treatment dilution. Keeping the ratio of carrier solvent constant allowed for comparisons between tested environmental samples; however, the YES/YAS does have a higher ratio of solvent compared to that used in the RTCA.

The highest dose of positive controls was set, where 17β-estradiol (E2) was 6.7e-9 M, 4-hydroxytamoxifen (4-HT) was 2.7e-6 M, 5α-dihydrotestosterone (DHT) was 6.7e-8 M, and flutamide (FL) was 6.7e-9 M, and the positive controls were serial diluted from these doses. Agonist media only contained essential nutrients, while antagonist media also contained 3.3e-10 M E2 for YES or 3.3e-9 M DHT for YAS as a baseline level of agonism. For antagonist plates, the ability of the positive control or sample to inhibit this baseline level of agonism was measured.

Instead of an Induction Ratio based on the vehicle control for agonists, a ‘Reduction Ratio (R_R)’ was calculated based on the agonist baseline control to screen for antagonists:

$$R_R = \frac{OD690\ agonist\ baseline}{OD690\ sample} * \frac{net\ absorbance\ sample\ (OD570 - 690)}{net\ absorbance\ agonist\ baseline\ (OD570 - 690)}$$

Where the optical densities (OD) were measured at both wavelengths of 570 nm (OD_{570}) and 690 nm (OD_{690}). OD_{690} measures yeast growth and also functions as a correctional value for diffraction when OD_{570} is measured for color development. With the R_R, a positive result is a decrease in induction from the agonist baseline control. R_R was normalized with 0% set as the highest dose of the antagonist hormone, FL or 4-HT, where the greatest degree of antagonism is known to occur, while 100% is the lowest dose of FL or 4-HT, which has negligible antagonism. Next, a non-linear fit with the formula of ‘log(inhibition) versus response’ was used.

The equivalence (EQ) factor to the corresponding positive controls for each assay were calculated to assess toxic potency:

$$Positive\ control\ equivalence = \frac{EC_{50\ sample}}{EC_{50\ positive\ control}}$$

Where $EC_{50\ positive\ control}$ is based on the dose-response curve of agonist (in the agonist plates) or antagonist (in the antagonist plates) positive control.

A preliminary cytotoxicity test of the samples was done to ensure that the doses were sub-lethal, ranging from 0.625× to 10× in duplicate with an exposure time from 18 to 46 h. Incorporating the cytotoxicity data, a half logarithmic dilution scheme was used, with the cytotoxic samples having the highest dose of 3× and the non-cytotoxic samples increasing to a dose of 10×.

RESULTS

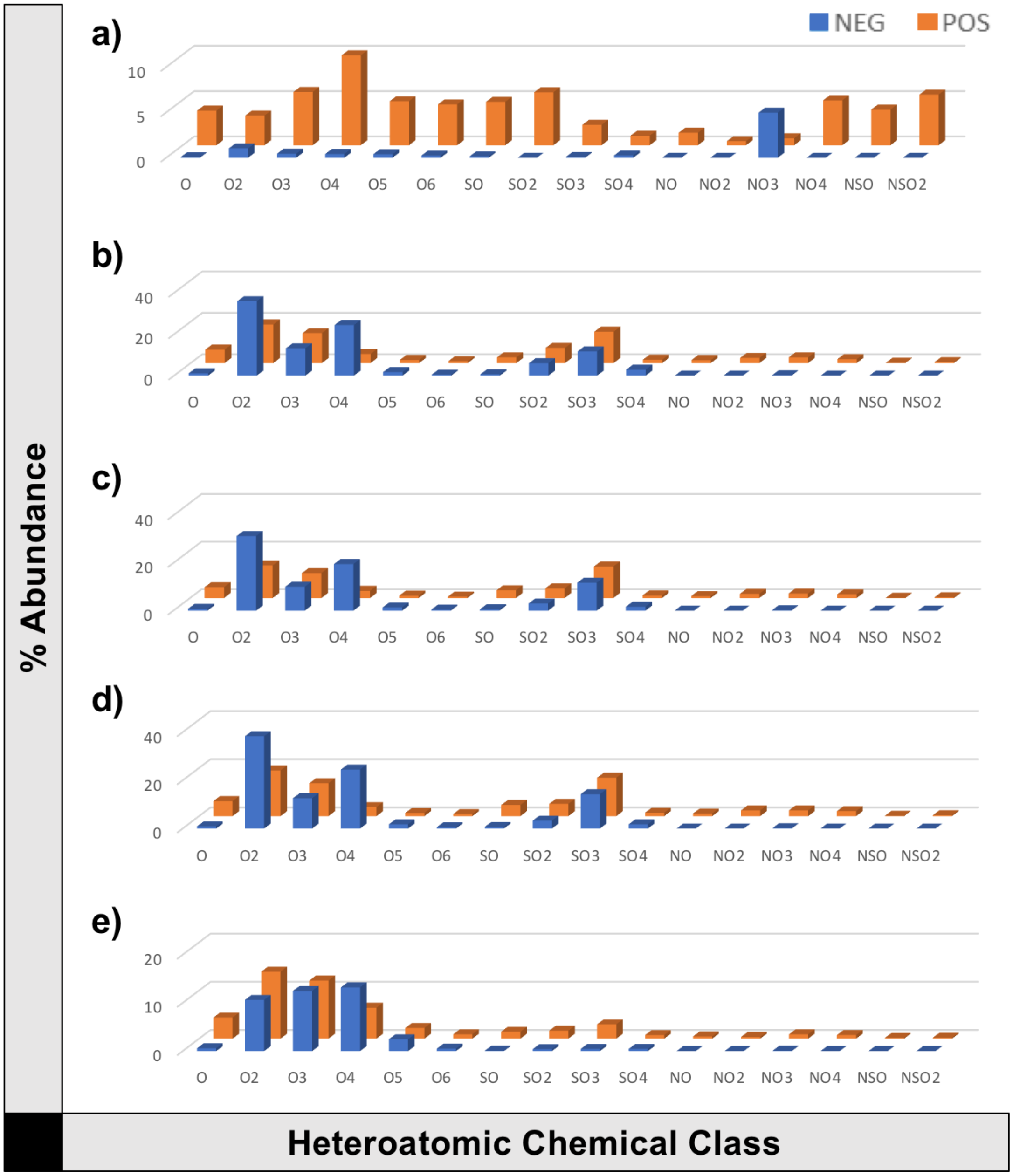

Figure S2: Heteroatomic chemical class distributions of environmental samples: a) River 2017, b) BML 2013, c) BML 2015, d) BML 2017, e) Pond 9 (2016). Blue (NEG) is negative ionization mode and orange (POS) is positive ionization mode. The % Abundance is calculated separately in each mode from the total intensity of selected chemical classes detected, excluding chemical classes that were not selected and signals without an assigned formula.

Table S1: Average ppm error of environmental sample extracts for formula assignment in high-resolution mass spectrometry analysis.

Environmental Sample	Ionization Mode	Average ppm Error per Analysis	Overall Average ppm Error
Pond 9 2016	Negative	0.80	
BML 2015	Negative	0.99	
BML 2017	Negative	1.02	
BML 2013	Negative	1.09	
River 2017	Negative	1.17	1.63
River 2017	Positive	2.15	
BML 2017	Positive	2.24	
Pond 9 2016	Positive	2.25	
BML 2015	Positive	2.27	
BML 2013	Positive	2.30	

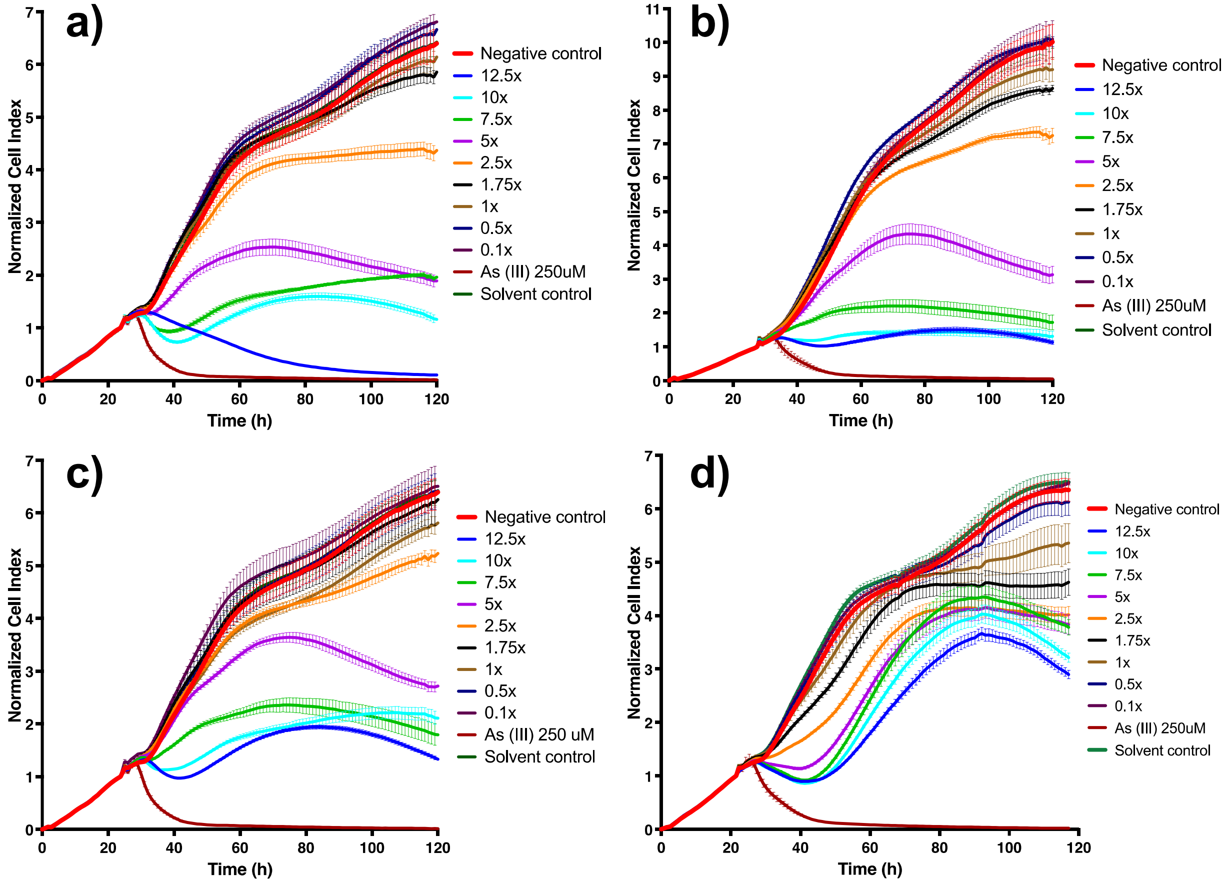

Figure S3: Toxicity response profiles of HepG2 cells after exposure to the extractable organics of a) BML 2013, b) BML 2015, c) BML 2017, and d) Pond 9 2016. Values are the mean ± SEM (n=4 replicates in a plate).

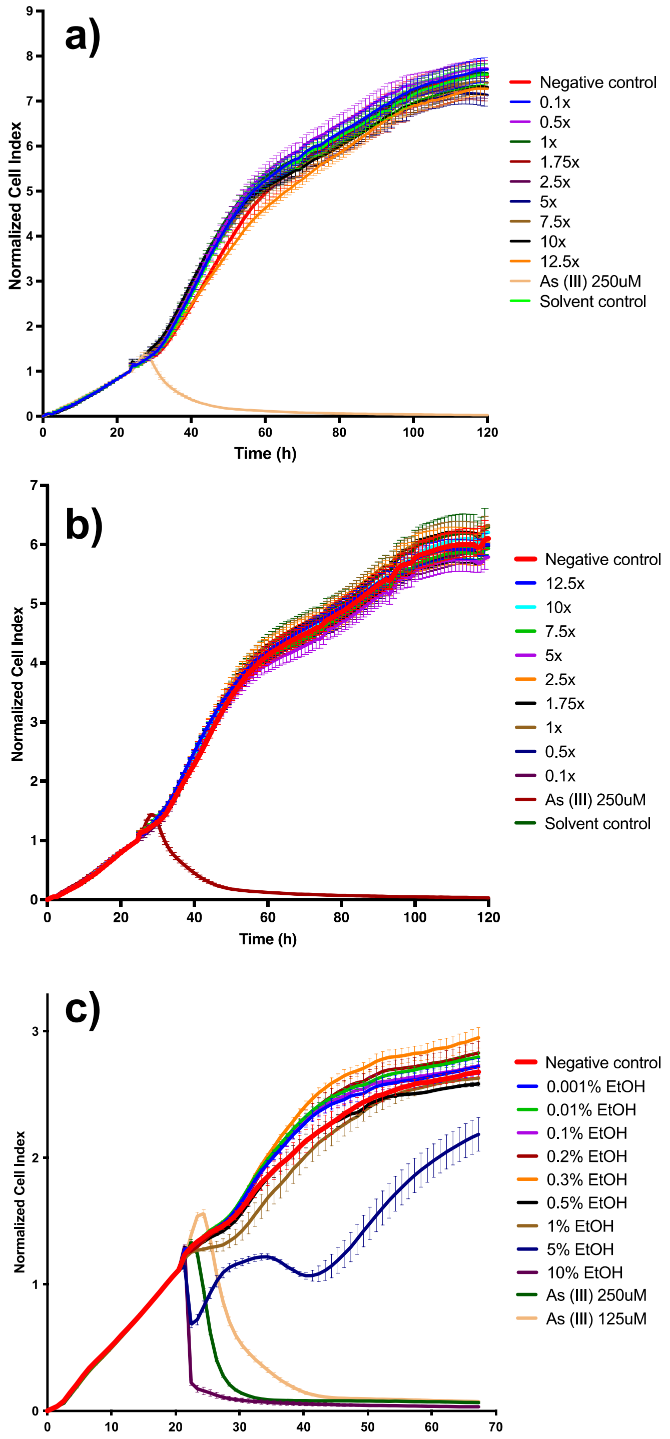

Figure S4: Toxicity response profiles of HepG2 cells after treatment with the extractable organics of a) River 2017 and b) LC/MS grade water, and with c) anhydrous ethanol (EtOH), and arsenite (As [III]). Values are the mean ± SEM (n=4 replicates within a plate).

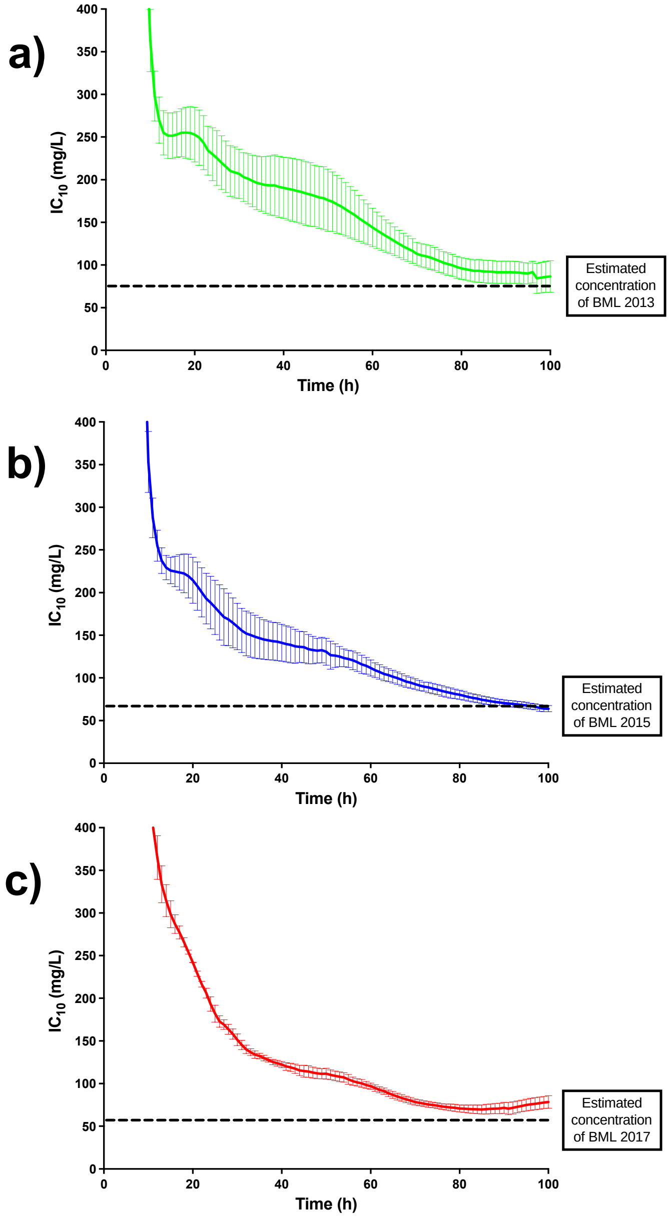

Figure S5: Threshold measurement (IC_{10}) over time of the extractable organics of a) BML 2013, b) BML 2015, and c) BML 2017, with their corresponding field concentration indicated by the dotted line. Values are the mean ± SEM (n=3) of plate replicates.

Endocrine activity. For flutamide EQ, the experimentally determined flutamide EC_{50} was used (2.89 mg/L), which was close to its theoretical EC_{50} (2.91 mg/L). For 4-hydroxytamoxifen EQ, the experimental 4-hydroxytamoxifen EC_{50} calculation was ambiguous and therefore its theoretical EC_{50} was used as a benchmark (1.23 mg/L).

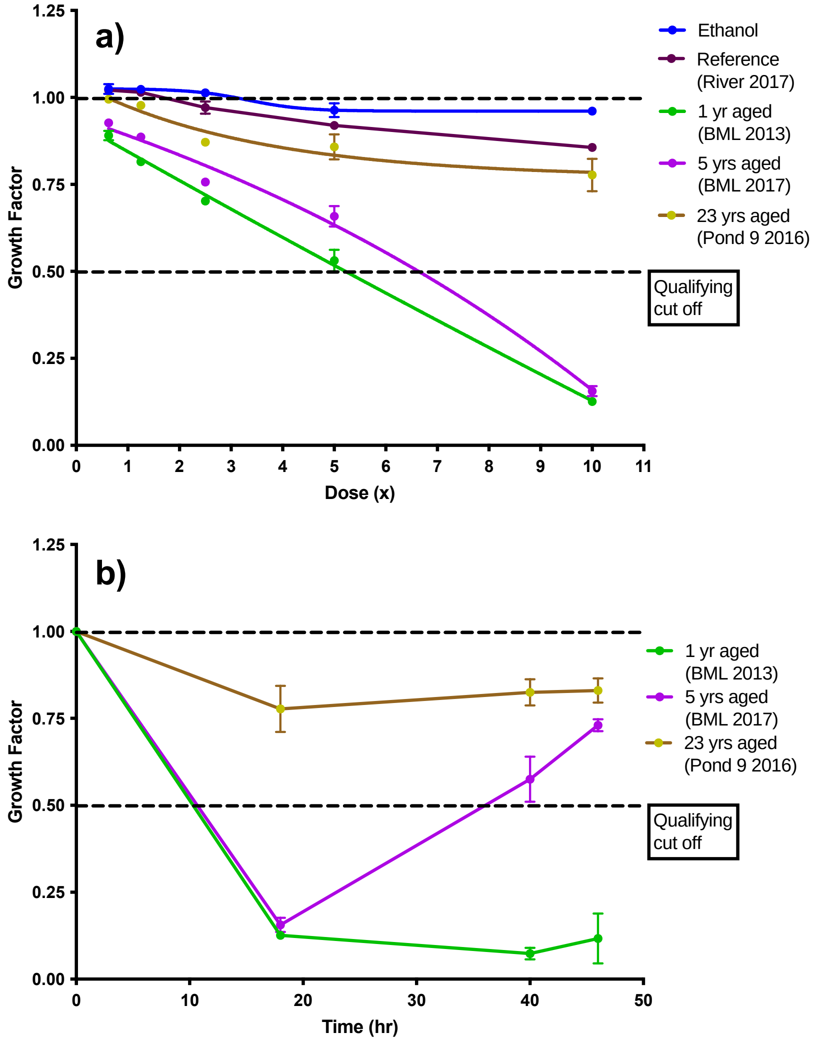

Figure S6: Growth of YES strain after treatments with extractable organics of environmental samples a) over a range of doses, and b) monitoring the 10× dose over time. Values are the mean ± SEM (n=2 replicates in a plate).

Table S2: Quantifying the antagonistic potency of environmental sample extracts by YES/YAS.

	Field mg/L	YES Antagonism			YAS Antagonism		
		EC_{50} (mg/L)	EC_{50} (×)	4-HT EQ	EC_{50} (mg/L)	EC_{50} (×)	FL EQ
**1 yr aged (BML 2013)**	75.8	1.27	0.016	1.03	16.3	0.22	5.64
**5 yrs aged (BML 2017)**	57.9	0.33	0.005	0.27	11.9	0.21	4.11
**23 yrs aged (Pond 9 2016)**	38.1	0.83	0.022	0.68	17.3	0.45	5.98
**Reference (River 2017)**	0.76	127	167	103	>30,000	>40,000	>10,000

Note: Field mg/L is the environmentally relevant field concentration based on gravimetric analysis of the extract. The River 2017 reference did not generate a full dose response for YAS antagonism and therefore the EC_{50} calculation was ambiguous, i.e., ">". 4-Hydroxytamoxifen (4-HT) and flutamide (FL) are known antagonistic chemicals and are compared to environmental samples to derive a positive control equivalence ratio (EQ), where an EQ less than 1 is indicative of greater potency than the given antagonistic chemical.

REFERENCES

1. Shotyk W, Bicalho B, Cuss CW, et al. Trace metals in the dissolved fraction (< 0.45 µm) of the lower Athabasca River: Analytical challenges and environmental implications. *Sci Total Environ*. 2017;580:660-669. doi:10.1016/j.scitotenv.2016.12.012
2. Moe B, Gabos S, Li X. Real-time cell-microelectronic sensing of nanoparticle-induced cytotoxic effects. *Anal Chim Acta*. 2013;789:83-90.
3. Li J, Moe B, Vemula S, Wang W, Li X-F. Emerging Disinfection Byproducts, Halobenzoquinones: Effects of Isomeric Structure and Halogen Substitution on Cytotoxicity, Formation of Reactive Oxygen Species, and Genotoxicity. *Environ Sci Technol*. 2016:acs.est.5b05585. doi:10.1021/acs.est.5b05585
4. Moe B. *Cell-electronic Sensing of Cellular Responses and Toxicity Induced by Nanoparticles and Arsenic Species*. Dissertation. University of Alberta; 2013.
5. Xenometrix. XenoScreen XL YES/YAS, short protocol, version 3.02. 2015.

STATISTICS ATTACHMENT

Table S3: ANOVA summary on an enrichment factor (×) scale for HepG2 cells exposed to OSPW extracts in the RTCA assay.

Source of Variation	% of total variation	P value	P value summary	Significant?
Interaction	20.74	<0.0001	****	Yes
Time	58.97	<0.0001	****	Yes
OSPW	9.926	<0.0001	****	Yes

Table S4: Tukey summary on an enrichment factor (×) scale for HepG2 cells exposed to OSPW extracts in the RTCA assay.

Tukey's multiple comparisons test	Mean Diff.	95.00% CI of diff.	Summary	Adjusted P Value
10				
BML 2017 vs. BML 2013	39.58	34.39 to 44.77	****	<0.0001
BML 2017 vs. Pond 9 2016	16.93	11.74 to 22.12	****	<0.0001
BML 2017 vs. BML 2015	21.53	16.34 to 26.72	****	<0.0001
BML 2013 vs. Pond 9 2016	-22.65	-27.84 to -17.46	****	<0.0001
BML 2013 vs. BML 2015	-18.05	-23.24 to -12.86	****	<0.0001
Pond 9 2016 vs. BML 2015	4.593	-0.5957 to 9.782	ns	0.1038
11				
BML 2017 vs. BML 2013	24.64	19.45 to 29.83	****	<0.0001
BML 2017 vs. Pond 9 2016	10.24	5.048 to 15.43	****	<0.0001
BML 2017 vs. BML 2015	13.36	8.171 to 18.55	****	<0.0001
BML 2013 vs. Pond 9 2016	-14.4	-19.59 to -9.211	****	<0.0001
BML 2013 vs. BML 2015	-11.28	-16.47 to -6.088	****	<0.0001
Pond 9 2016 vs. BML 2015	3.123	-2.066 to 8.312	ns	0.4082
12				
BML 2017 vs. BML 2013	18.34	13.15 to 23.53	****	<0.0001
BML 2017 vs. Pond 9 2016	8.323	3.134 to 13.51	***	0.0002
BML 2017 vs. BML 2015	10.15	4.961 to 15.34	****	<0.0001

Tukey's multiple comparisons test	Mean Diff.	95.00% CI of diff.	Summary	Adjusted P Value
BML 2013 vs. Pond 9 2016	-10.01	-15.20 to -4.824	****	<0.0001
BML 2013 vs. BML 2015	-8.187	-13.38 to -2.998	***	0.0003
Pond 9 2016 vs. BML 2015	1.827	-3.362 to 7.016	ns	0.8014
13				
BML 2017 vs. BML 2013	14.16	8.971 to 19.35	****	<0.0001
BML 2017 vs. Pond 9 2016	7.19	2.001 to 12.38	**	0.0022
BML 2017 vs. BML 2015	7.757	2.568 to 12.95	***	0.0007
BML 2013 vs. Pond 9 2016	-6.97	-12.16 to -1.781	**	0.0032
BML 2013 vs. BML 2015	-6.403	-11.59 to -1.214	**	0.0084
Pond 9 2016 vs. BML 2015	0.5667	-4.622 to 5.756	ns	0.9922
14				
BML 2017 vs. BML 2013	11.94	6.749 to 17.13	****	<0.0001
BML 2017 vs. Pond 9 2016	7.1	1.911 to 12.29	**	0.0026
BML 2017 vs. BML 2015	6.603	1.414 to 11.79	**	0.006
BML 2013 vs. Pond 9 2016	-4.838	-10.03 to 0.3514	ns	0.0778
BML 2013 vs. BML 2015	-5.334	-10.52 to -0.1453	*	0.0412
Pond 9 2016 vs. BML 2015	-0.4967	-5.686 to 4.692	ns	0.9947
15				
BML 2017 vs. BML 2013	10.31	5.125 to 15.50	****	<0.0001
BML 2017 vs. Pond 9 2016	7.14	1.951 to 12.33	**	0.0024
BML 2017 vs. BML 2015	5.633	0.4443 to 10.82	*	0.0272
BML 2013 vs. Pond 9 2016	-3.174	-8.363 to 2.015	ns	0.3934
BML 2013 vs. BML 2015	-4.681	-9.870 to 0.5084	ns	0.0938
Pond 9 2016 vs. BML 2015	-1.506	-6.695 to 3.683	ns	0.8777
16				
BML 2017 vs. BML 2013	9.267	4.078 to 14.46	****	<0.0001
BML 2017 vs. Pond 9 2016	7.422	2.233 to 12.61	**	0.0014
BML 2017 vs. BML 2015	5.083	-0.1057 to 10.27	ns	0.0573
BML 2013 vs. Pond 9 2016	-1.844	-7.033 to 3.345	ns	0.7967
BML 2013 vs. BML 2015	-4.183	-9.372 to 1.006	ns	0.1619
Pond 9 2016 vs. BML 2015	-2.339	-7.528 to 2.850	ns	0.6518
17				

Tukey's multiple comparisons test	Mean Diff.	95.00% CI of diff.	Summary	Adjusted P Value
BML 2017 vs. BML 2013	8.381	3.192 to 13.57	***	0.0002
BML 2017 vs. Pond 9 2016	7.585	2.396 to 12.77	**	0.001
BML 2017 vs. BML 2015	4.6	-0.5891 to 9.789	ns	0.103
BML 2013 vs. Pond 9 2016	-0.796	-5.985 to 4.393	ns	0.9791
BML 2013 vs. BML 2015	-3.781	-8.970 to 1.408	ns	0.2392
Pond 9 2016 vs. BML 2015	-2.985	-8.174 to 2.204	ns	0.4494
18				
BML 2017 vs. BML 2013	7.513	2.324 to 12.70	**	0.0012
BML 2017 vs. Pond 9 2016	7.554	2.365 to 12.74	**	0.0011
BML 2017 vs. BML 2015	4.063	-1.126 to 9.252	ns	0.1828
BML 2013 vs. Pond 9 2016	0.04033	-5.149 to 5.229	ns	>0.9999
BML 2013 vs. BML 2015	-3.45	-8.639 to 1.739	ns	0.318
Pond 9 2016 vs. BML 2015	-3.49	-8.679 to 1.699	ns	0.3077
19				
BML 2017 vs. BML 2013	6.814	1.625 to 12.00	**	0.0042
BML 2017 vs. Pond 9 2016	7.509	2.320 to 12.70	**	0.0012
BML 2017 vs. BML 2015	3.666	-1.523 to 8.855	ns	0.2651
BML 2013 vs. Pond 9 2016	0.695	-4.494 to 5.884	ns	0.9859
BML 2013 vs. BML 2015	-3.148	-8.337 to 2.041	ns	0.4009
Pond 9 2016 vs. BML 2015	-3.843	-9.032 to 1.346	ns	0.2258
20				
BML 2017 vs. BML 2013	6.188	0.9993 to 11.38	*	0.0119
BML 2017 vs. Pond 9 2016	7.367	2.178 to 12.56	**	0.0016
BML 2017 vs. BML 2015	3.375	-1.814 to 8.564	ns	0.3377
BML 2013 vs. Pond 9 2016	1.178	-4.011 to 6.367	ns	0.9367
BML 2013 vs. BML 2015	-2.813	-8.002 to 2.376	ns	0.5022
Pond 9 2016 vs. BML 2015	-3.991	-9.180 to 1.198	ns	0.1962
21				
BML 2017 vs. BML 2013	5.591	0.4019 to 10.78	*	0.0289
BML 2017 vs. Pond 9 2016	7.115	1.926 to 12.30	**	0.0025
BML 2017 vs. BML 2015	3.095	-2.094 to 8.284	ns	0.4165
BML 2013 vs. Pond 9 2016	1.524	-3.665 to 6.713	ns	0.8739

Tukey's multiple comparisons test	Mean Diff.	95.00% CI of diff.	Summary	Adjusted P Value
BML 2013 vs. BML 2015	-2.496	-7.685 to 2.693	ns	0.6024
Pond 9 2016 vs. BML 2015	-4.02	-9.209 to 1.169	ns	0.1908
22				
BML 2017 vs. BML 2013	5.047	-0.1424 to 10.24	ns	0.0601
BML 2017 vs. Pond 9 2016	6.781	1.592 to 11.97	**	0.0045
BML 2017 vs. BML 2015	2.814	-2.375 to 8.003	ns	0.5018
BML 2013 vs. Pond 9 2016	1.735	-3.454 to 6.924	ns	0.825
BML 2013 vs. BML 2015	-2.232	-7.421 to 2.957	ns	0.6848
Pond 9 2016 vs. BML 2015	-3.967	-9.156 to 1.222	ns	0.2009
23				
BML 2017 vs. BML 2013	4.688	-0.5011 to 9.877	ns	0.093
BML 2017 vs. Pond 9 2016	6.558	1.369 to 11.75	**	0.0065
BML 2017 vs. BML 2015	2.631	-2.558 to 7.820	ns	0.5595
BML 2013 vs. Pond 9 2016	1.87	-3.319 to 7.059	ns	0.7898
BML 2013 vs. BML 2015	-2.057	-7.246 to 3.132	ns	0.7374
Pond 9 2016 vs. BML 2015	-3.927	-9.116 to 1.262	ns	0.2088
24				
BML 2017 vs. BML 2013	4.248	-0.9411 to 9.437	ns	0.1514
BML 2017 vs. Pond 9 2016	6.182	0.9929 to 11.37	*	0.012
BML 2017 vs. BML 2015	2.314	-2.875 to 7.503	ns	0.6595
BML 2013 vs. Pond 9 2016	1.934	-3.255 to 7.123	ns	0.7723
BML 2013 vs. BML 2015	-1.934	-7.123 to 3.255	ns	0.7724
Pond 9 2016 vs. BML 2015	-3.868	-9.057 to 1.321	ns	0.2208
25				
BML 2017 vs. BML 2013	3.889	-1.300 to 9.078	ns	0.2164
BML 2017 vs. Pond 9 2016	5.82	0.6309 to 11.01	*	0.0208
BML 2017 vs. BML 2015	2.089	-3.100 to 7.278	ns	0.7279
BML 2013 vs. Pond 9 2016	1.931	-3.258 to 7.120	ns	0.7731
BML 2013 vs. BML 2015	-1.8	-6.989 to 3.389	ns	0.8083
Pond 9 2016 vs. BML 2015	-3.731	-8.920 to 1.458	ns	0.2502
26				
BML 2017 vs. BML 2013	3.537	-1.652 to 8.726	ns	0.296

Tukey's multiple comparisons test	Mean Diff.	95.00% CI of diff.	Summary	Adjusted P Value
BML 2017 vs. Pond 9 2016	5.413	0.2243 to 10.60	*	0.037
BML 2017 vs. BML 2015	1.862	-3.327 to 7.051	ns	0.7921
BML 2013 vs. Pond 9 2016	1.876	-3.313 to 7.065	ns	0.7881
BML 2013 vs. BML 2015	-1.675	-6.864 to 3.514	ns	0.8395
Pond 9 2016 vs. BML 2015	-3.552	-8.741 to 1.637	ns	0.2924
27				
BML 2017 vs. BML 2013	3.379	-1.810 to 8.568	ns	0.3367
BML 2017 vs. Pond 9 2016	5.164	-0.02540 to 10.35	ns	0.0517
BML 2017 vs. BML 2015	1.832	-3.357 to 7.021	ns	0.8
BML 2013 vs. Pond 9 2016	1.785	-3.404 to 6.974	ns	0.8123
BML 2013 vs. BML 2015	-1.547	-6.736 to 3.642	ns	0.8689
Pond 9 2016 vs. BML 2015	-3.332	-8.521 to 1.857	ns	0.3493
28				
BML 2017 vs. BML 2013	3.216	-1.973 to 8.405	ns	0.3814
BML 2017 vs. Pond 9 2016	4.854	-0.3347 to 10.04	ns	0.0763
BML 2017 vs. BML 2015	1.712	-3.477 to 6.901	ns	0.8307
BML 2013 vs. Pond 9 2016	1.638	-3.551 to 6.827	ns	0.8484
BML 2013 vs. BML 2015	-1.505	-6.694 to 3.684	ns	0.8781
Pond 9 2016 vs. BML 2015	-3.143	-8.332 to 2.046	ns	0.4025
29				
BML 2017 vs. BML 2013	3.04	-2.149 to 8.229	ns	0.4327
BML 2017 vs. Pond 9 2016	4.531	-0.6577 to 9.720	ns	0.1113
BML 2017 vs. BML 2015	1.618	-3.571 to 6.807	ns	0.853
BML 2013 vs. Pond 9 2016	1.491	-3.698 to 6.680	ns	0.8809
BML 2013 vs. BML 2015	-1.422	-6.611 to 3.767	ns	0.8948
Pond 9 2016 vs. BML 2015	-2.913	-8.102 to 2.276	ns	0.4713
30				
BML 2017 vs. BML 2013	2.879	-2.310 to 8.068	ns	0.4817
BML 2017 vs. Pond 9 2016	4.196	-0.9934 to 9.385	ns	0.1599
BML 2017 vs. BML 2015	1.57	-3.619 to 6.759	ns	0.8638
BML 2013 vs. Pond 9 2016	1.317	-3.872 to 6.506	ns	0.9143
BML 2013 vs. BML 2015	-1.309	-6.498 to 3.880	ns	0.9157

Tukey's multiple comparisons test	Mean Diff.	95.00% CI of diff.	Summary	Adjusted P Value
Pond 9 2016 vs. BML 2015	-2.625	-7.814 to 2.564	ns	0.5614
31				
BML 2017 vs. BML 2013	2.755	-2.434 to 7.944	ns	0.5204
BML 2017 vs. Pond 9 2016	3.849	-1.340 to 9.038	ns	0.2247
BML 2017 vs. BML 2015	1.547	-3.642 to 6.736	ns	0.8689
BML 2013 vs. Pond 9 2016	1.094	-4.095 to 6.283	ns	0.9485
BML 2013 vs. BML 2015	-1.208	-6.397 to 3.981	ns	0.9322
Pond 9 2016 vs. BML 2015	-2.301	-7.490 to 2.888	ns	0.6635
32				
BML 2017 vs. BML 2013	2.63	-2.559 to 7.819	ns	0.5599
BML 2017 vs. Pond 9 2016	3.447	-1.742 to 8.636	ns	0.3187
BML 2017 vs. BML 2015	1.499	-3.690 to 6.688	ns	0.8793
BML 2013 vs. Pond 9 2016	0.8173	-4.372 to 6.006	ns	0.9774
BML 2013 vs. BML 2015	-1.131	-6.320 to 4.058	ns	0.9434
Pond 9 2016 vs. BML 2015	-1.949	-7.138 to 3.240	ns	0.7682
33				
BML 2017 vs. BML 2013	2.524	-2.665 to 7.713	ns	0.5934
BML 2017 vs. Pond 9 2016	3.047	-2.142 to 8.236	ns	0.4307
BML 2017 vs. BML 2015	1.427	-3.762 to 6.616	ns	0.8939
BML 2013 vs. Pond 9 2016	0.5227	-4.666 to 5.712	ns	0.9939
BML 2013 vs. BML 2015	-1.097	-6.286 to 4.092	ns	0.948
Pond 9 2016 vs. BML 2015	-1.62	-6.809 to 3.569	ns	0.8526
34				
BML 2017 vs. BML 2013	2.459	-2.730 to 7.648	ns	0.6141
BML 2017 vs. Pond 9 2016	2.641	-2.548 to 7.830	ns	0.5564
BML 2017 vs. BML 2015	1.402	-3.787 to 6.591	ns	0.8986
BML 2013 vs. Pond 9 2016	0.182	-5.007 to 5.371	ns	0.9997
BML 2013 vs. BML 2015	-1.057	-6.246 to 4.132	ns	0.9532
Pond 9 2016 vs. BML 2015	-1.239	-6.428 to 3.950	ns	0.9274
35				
BML 2017 vs. BML 2013	2.407	-2.782 to 7.596	ns	0.6305
BML 2017 vs. Pond 9 2016	2.213	-2.976 to 7.402	ns	0.6908

Tukey's multiple comparisons test	Mean Diff.	95.00% CI of diff.	Summary	Adjusted P Value
BML 2017 vs. BML 2015	1.394	-3.795 to 6.583	ns	0.9002
BML 2013 vs. Pond 9 2016	-0.1943	-5.383 to 4.995	ns	0.9997
BML 2013 vs. BML 2015	-1.013	-6.202 to 4.176	ns	0.9585
Pond 9 2016 vs. BML 2015	-0.8183	-6.007 to 4.371	ns	0.9774
36				
BML 2017 vs. BML 2013	2.34	-2.849 to 7.529	ns	0.6515
BML 2017 vs. Pond 9 2016	1.757	-3.432 to 6.946	ns	0.8195
BML 2017 vs. BML 2015	1.37	-3.819 to 6.559	ns	0.9047
BML 2013 vs. Pond 9 2016	-0.5833	-5.772 to 4.606	ns	0.9915
BML 2013 vs. BML 2015	-0.9697	-6.159 to 4.219	ns	0.9632
Pond 9 2016 vs. BML 2015	-0.3863	-5.575 to 4.803	ns	0.9975
37				
BML 2017 vs. BML 2013	2.284	-2.905 to 7.473	ns	0.6689
BML 2017 vs. Pond 9 2016	1.258	-3.931 to 6.447	ns	0.9243
BML 2017 vs. BML 2015	1.356	-3.833 to 6.545	ns	0.9073
BML 2013 vs. Pond 9 2016	-1.026	-6.215 to 4.163	ns	0.9569
BML 2013 vs. BML 2015	-0.928	-6.117 to 4.261	ns	0.9675
Pond 9 2016 vs. BML 2015	0.098	-5.091 to 5.287	ns	>0.9999
38				
BML 2017 vs. BML 2013	2.242	-2.947 to 7.431	ns	0.6818
BML 2017 vs. Pond 9 2016	0.7473	-4.442 to 5.936	ns	0.9826
BML 2017 vs. BML 2015	1.355	-3.834 to 6.544	ns	0.9076
BML 2013 vs. Pond 9 2016	-1.495	-6.684 to 3.694	ns	0.8802
BML 2013 vs. BML 2015	-0.8873	-6.076 to 4.302	ns	0.9714
Pond 9 2016 vs. BML 2015	0.6073	-4.582 to 5.796	ns	0.9905
39				
BML 2017 vs. BML 2013	2.236	-2.953 to 7.425	ns	0.6838
BML 2017 vs. Pond 9 2016	0.253	-4.936 to 5.442	ns	0.9993
BML 2017 vs. BML 2015	1.359	-3.830 to 6.548	ns	0.9068
BML 2013 vs. Pond 9 2016	-1.983	-7.172 to 3.206	ns	0.7587
BML 2013 vs. BML 2015	-0.8767	-6.066 to 4.312	ns	0.9724
Pond 9 2016 vs. BML 2015	1.106	-4.083 to 6.295	ns	0.9468

Tukey's multiple comparisons test	Mean Diff.	95.00% CI of diff.	Summary	Adjusted P Value
40				
BML 2017 vs. BML 2013	2.217	-2.972 to 7.406	ns	0.6894
BML 2017 vs. Pond 9 2016	-0.3027	-5.492 to 4.886	ns	0.9988
BML 2017 vs. BML 2015	1.379	-3.810 to 6.568	ns	0.903
BML 2013 vs. Pond 9 2016	-2.52	-7.709 to 2.669	ns	0.5948
BML 2013 vs. BML 2015	-0.838	-6.027 to 4.351	ns	0.9758
Pond 9 2016 vs. BML 2015	1.682	-3.507 to 6.871	ns	0.8379
41				
BML 2017 vs. BML 2013	2.177	-3.012 to 7.366	ns	0.7015
BML 2017 vs. Pond 9 2016	-0.9133	-6.102 to 4.276	ns	0.969
BML 2017 vs. BML 2015	1.365	-3.824 to 6.554	ns	0.9056
BML 2013 vs. Pond 9 2016	-3.091	-8.280 to 2.098	ns	0.4177
BML 2013 vs. BML 2015	-0.812	-6.001 to 4.377	ns	0.9779
Pond 9 2016 vs. BML 2015	2.279	-2.910 to 7.468	ns	0.6706
42				
BML 2017 vs. BML 2013	2.174	-3.015 to 7.363	ns	0.7026
BML 2017 vs. Pond 9 2016	-1.505	-6.694 to 3.684	ns	0.8781
BML 2017 vs. BML 2015	1.385	-3.804 to 6.574	ns	0.9019
BML 2013 vs. Pond 9 2016	-3.678	-8.867 to 1.511	ns	0.2622
BML 2013 vs. BML 2015	-0.7883	-5.977 to 4.401	ns	0.9797
Pond 9 2016 vs. BML 2015	2.89	-2.299 to 8.079	ns	0.4783
43				
BML 2017 vs. BML 2013	2.156	-3.033 to 7.345	ns	0.708
BML 2017 vs. Pond 9 2016	-2.117	-7.306 to 3.072	ns	0.7197
BML 2017 vs. BML 2015	1.409	-3.780 to 6.598	ns	0.8974
BML 2013 vs. Pond 9 2016	-4.273	-9.462 to 0.9164	ns	0.1476
BML 2013 vs. BML 2015	-0.7473	-5.936 to 4.442	ns	0.9826
Pond 9 2016 vs. BML 2015	3.525	-1.664 to 8.714	ns	0.2989
44				
BML 2017 vs. BML 2013	2.12	-3.069 to 7.309	ns	0.7186
BML 2017 vs. Pond 9 2016	-2.777	-7.966 to 2.412	ns	0.5136
BML 2017 vs. BML 2015	1.379	-3.810 to 6.568	ns	0.903

Tukey's multiple comparisons test	Mean Diff.	95.00% CI of diff.	Summary	Adjusted P Value
BML 2013 vs. Pond 9 2016	-4.897	-10.09 to 0.2921	ns	0.0724
BML 2013 vs. BML 2015	-0.741	-5.930 to 4.448	ns	0.983
Pond 9 2016 vs. BML 2015	4.156	-1.033 to 9.345	ns	0.1665
45				
BML 2017 vs. BML 2013	2.118	-3.071 to 7.307	ns	0.7193
BML 2017 vs. Pond 9 2016	-3.356	-8.545 to 1.833	ns	0.3427
BML 2017 vs. BML 2015	1.383	-3.806 to 6.572	ns	0.9024
BML 2013 vs. Pond 9 2016	-5.474	-10.66 to -0.2853	*	0.0341
BML 2013 vs. BML 2015	-0.7353	-5.924 to 4.454	ns	0.9834
Pond 9 2016 vs. BML 2015	4.739	-0.4501 to 9.928	ns	0.0876
46				
BML 2017 vs. BML 2013	2.108	-3.081 to 7.297	ns	0.7222
BML 2017 vs. Pond 9 2016	-3.98	-9.169 to 1.209	ns	0.1983
BML 2017 vs. BML 2015	1.417	-3.772 to 6.606	ns	0.8959
BML 2013 vs. Pond 9 2016	-6.089	-11.28 to -0.8996	*	0.0138
BML 2013 vs. BML 2015	-0.6917	-5.881 to 4.497	ns	0.9861
Pond 9 2016 vs. BML 2015	5.397	0.2079 to 10.59	*	0.0379
47				
BML 2017 vs. BML 2013	2.075	-3.114 to 7.264	ns	0.732
BML 2017 vs. Pond 9 2016	-4.951	-10.14 to 0.2381	ns	0.0677
BML 2017 vs. BML 2015	1.425	-3.764 to 6.614	ns	0.8942
BML 2013 vs. Pond 9 2016	-7.026	-12.22 to -1.837	**	0.0029
BML 2013 vs. BML 2015	-0.65	-5.839 to 4.539	ns	0.9884
Pond 9 2016 vs. BML 2015	6.376	1.187 to 11.57	**	0.0088
48				
BML 2017 vs. BML 2013	2.061	-3.128 to 7.250	ns	0.7361
BML 2017 vs. Pond 9 2016	-5.421	-10.61 to -0.2319	*	0.0367
BML 2017 vs. BML 2015	1.423	-3.766 to 6.612	ns	0.8947
BML 2013 vs. Pond 9 2016	-7.482	-12.67 to -2.293	**	0.0013
BML 2013 vs. BML 2015	-0.6383	-5.827 to 4.551	ns	0.989
Pond 9 2016 vs. BML 2015	6.844	1.655 to 12.03	**	0.004
49				

Tukey's multiple comparisons test	Mean Diff.	95.00% CI of diff.	Summary	Adjusted P Value
BML 2017 vs. BML 2013	2.044	-3.145 to 7.233	ns	0.741
BML 2017 vs. Pond 9 2016	-5.824	-11.01 to -0.6346	*	0.0207
BML 2017 vs. BML 2015	1.388	-3.801 to 6.577	ns	0.9014
BML 2013 vs. Pond 9 2016	-7.868	-13.06 to -2.679	***	0.0006
BML 2013 vs. BML 2015	-0.6567	-5.846 to 4.532	ns	0.988
Pond 9 2016 vs. BML 2015	7.211	2.022 to 12.40	**	0.0021
50				
BML 2017 vs. BML 2013	2.059	-3.130 to 7.248	ns	0.7367
BML 2017 vs. Pond 9 2016	-6.327	-11.52 to -1.138	**	0.0095
BML 2017 vs. BML 2015	1.426	-3.763 to 6.615	ns	0.8941
BML 2013 vs. Pond 9 2016	-8.386	-13.58 to -3.197	***	0.0002
BML 2013 vs. BML 2015	-0.633	-5.822 to 4.556	ns	0.9893
Pond 9 2016 vs. BML 2015	7.753	2.564 to 12.94	***	0.0007
51				
BML 2017 vs. BML 2013	2.012	-3.177 to 7.201	ns	0.7502
BML 2017 vs. Pond 9 2016	-7.104	-12.29 to -1.915	**	0.0025
BML 2017 vs. BML 2015	1.45	-3.739 to 6.639	ns	0.8893
BML 2013 vs. Pond 9 2016	-9.116	-14.31 to -3.927	****	<0.0001
BML 2013 vs. BML 2015	-0.5623	-5.751 to 4.627	ns	0.9924
Pond 9 2016 vs. BML 2015	8.554	3.365 to 13.74	***	0.0001
52				
BML 2017 vs. BML 2013	1.96	-3.229 to 7.149	ns	0.7651
BML 2017 vs. Pond 9 2016	-7.476	-12.67 to -2.287	**	0.0013
BML 2017 vs. BML 2015	1.391	-3.798 to 6.580	ns	0.9007
BML 2013 vs. Pond 9 2016	-9.436	-14.62 to -4.247	****	<0.0001
BML 2013 vs. BML 2015	-0.5683	-5.757 to 4.621	ns	0.9922
Pond 9 2016 vs. BML 2015	8.867	3.678 to 14.06	****	<0.0001
53				
BML 2017 vs. BML 2013	1.931	-3.258 to 7.120	ns	0.7731
BML 2017 vs. Pond 9 2016	-7.876	-13.06 to -2.687	***	0.0006
BML 2017 vs. BML 2015	1.384	-3.805 to 6.573	ns	0.9021
BML 2013 vs. Pond 9 2016	-9.807	-15.00 to -4.618	****	<0.0001

Tukey's multiple comparisons test	Mean Diff.	95.00% CI of diff.	Summary	Adjusted P Value
BML 2013 vs. BML 2015	-0.547	-5.736 to 4.642	ns	0.993
Pond 9 2016 vs. BML 2015	9.26	4.071 to 14.45	****	<0.0001
54				
BML 2017 vs. BML 2013	1.935	-3.254 to 7.124	ns	0.7719
BML 2017 vs. Pond 9 2016	-8.32	-13.51 to -3.131	***	0.0002
BML 2017 vs. BML 2015	1.409	-3.780 to 6.598	ns	0.8974
BML 2013 vs. Pond 9 2016	-10.26	-15.44 to -5.066	****	<0.0001
BML 2013 vs. BML 2015	-0.5263	-5.715 to 4.663	ns	0.9938
Pond 9 2016 vs. BML 2015	9.729	4.540 to 14.92	****	<0.0001
55				
BML 2017 vs. BML 2013	1.891	-3.298 to 7.080	ns	0.7842
BML 2017 vs. Pond 9 2016	-8.802	-13.99 to -3.613	****	<0.0001
BML 2017 vs. BML 2015	1.364	-3.825 to 6.553	ns	0.9058
BML 2013 vs. Pond 9 2016	-10.69	-15.88 to -5.504	****	<0.0001
BML 2013 vs. BML 2015	-0.5263	-5.715 to 4.663	ns	0.9938
Pond 9 2016 vs. BML 2015	10.17	4.978 to 15.36	****	<0.0001
56				
BML 2017 vs. BML 2013	1.84	-3.349 to 7.029	ns	0.7978
BML 2017 vs. Pond 9 2016	-9.277	-14.47 to -4.088	****	<0.0001
BML 2017 vs. BML 2015	1.329	-3.860 to 6.518	ns	0.9122
BML 2013 vs. Pond 9 2016	-11.12	-16.31 to -5.928	****	<0.0001
BML 2013 vs. BML 2015	-0.511	-5.700 to 4.678	ns	0.9943
Pond 9 2016 vs. BML 2015	10.61	5.417 to 15.80	****	<0.0001
57				
BML 2017 vs. BML 2013	1.826	-3.363 to 7.015	ns	0.8015
BML 2017 vs. Pond 9 2016	-9.584	-14.77 to -4.395	****	<0.0001
BML 2017 vs. BML 2015	1.318	-3.871 to 6.507	ns	0.9142
BML 2013 vs. Pond 9 2016	-11.41	-16.60 to -6.222	****	<0.0001
BML 2013 vs. BML 2015	-0.5087	-5.698 to 4.680	ns	0.9944
Pond 9 2016 vs. BML 2015	10.9	5.713 to 16.09	****	<0.0001
58				
BML 2017 vs. BML 2013	1.805	-3.384 to 6.994	ns	0.807

Tukey's multiple comparisons test	Mean Diff.	95.00% CI of diff.	Summary	Adjusted P Value
BML 2017 vs. Pond 9 2016	-9.838	-15.03 to -4.649	****	<0.0001
BML 2017 vs. BML 2015	1.314	-3.875 to 6.503	ns	0.9149
BML 2013 vs. Pond 9 2016	-11.64	-16.83 to -6.454	****	<0.0001
BML 2013 vs. BML 2015	-0.4913	-5.680 to 4.698	ns	0.9949
Pond 9 2016 vs. BML 2015	11.15	5.963 to 16.34	****	<0.0001
59				
BML 2017 vs. BML 2013	1.777	-3.412 to 6.966	ns	0.8142
BML 2017 vs. Pond 9 2016	-9.977	-15.17 to -4.788	****	<0.0001
BML 2017 vs. BML 2015	1.299	-3.890 to 6.488	ns	0.9175
BML 2013 vs. Pond 9 2016	-11.75	-16.94 to -6.565	****	<0.0001
BML 2013 vs. BML 2015	-0.4787	-5.668 to 4.710	ns	0.9953
Pond 9 2016 vs. BML 2015	11.28	6.087 to 16.46	****	<0.0001
60				
BML 2017 vs. BML 2013	1.742	-3.447 to 6.931	ns	0.8231
BML 2017 vs. Pond 9 2016	-10.09	-15.27 to -4.897	****	<0.0001
BML 2017 vs. BML 2015	1.274	-3.915 to 6.463	ns	0.9216
BML 2013 vs. Pond 9 2016	-11.83	-17.02 to -6.639	****	<0.0001
BML 2013 vs. BML 2015	-0.4677	-5.657 to 4.721	ns	0.9956
Pond 9 2016 vs. BML 2015	11.36	6.171 to 16.55	****	<0.0001

Table S5: ANOVA summary on a mg extract/L scale for HepG2 cells exposed to OSPW extracts in the RTCA assay.

Source of Variation	% of total variation	P value	P value summary	Significant?
Interaction	38.64	<0.0001	****	Yes
Time	38.87	<0.0001	****	Yes
OSPW	1.204	<0.0001	****	Yes

Table S6: Tukey summary on an mg extract/L scale for HepG2 cells exposed to OSPW extracts in the RTCA assay.

Tukey's multiple comparisons test	Mean Diff.	95.00% CI of diff.	Summary	Adjusted P Value
10				
Pond 9 2016 vs. BML 2013	-372.5	-678.2 to -66.90	**	0.0096
Pond 9 2016 vs. BML 2017	-19	-324.6 to 286.6	ns	0.9985
Pond 9 2016 vs. BML 2015	-341.2	-646.8 to -35.57	*	0.0217
BML 2013 vs. BML 2017	353.5	47.90 to 659.2	*	0.0158
BML 2013 vs. BML 2015	31.33	-274.3 to 337.0	ns	0.9935
BML 2017 vs. BML 2015	-322.2	-627.8 to -16.57	*	0.0343
11				
Pond 9 2016 vs. BML 2013	-248.7	-554.4 to 56.90	ns	0.1553
Pond 9 2016 vs. BML 2017	-43.37	-349.0 to 262.3	ns	0.9833
Pond 9 2016 vs. BML 2015	-202.7	-508.4 to 102.9	ns	0.3198
BML 2013 vs. BML 2017	205.4	-100.3 to 511.0	ns	0.3083
BML 2013 vs. BML 2015	46	-259.6 to 351.6	ns	0.9802
BML 2017 vs. BML 2015	-159.4	-465.0 to 146.3	ns	0.5355
12				
Pond 9 2016 vs. BML 2013	-224.5	-530.1 to 81.13	ns	0.2323
Pond 9 2016 vs. BML 2017	-101.7	-407.3 to 203.9	ns	0.8267
Pond 9 2016 vs. BML 2015	-180	-485.7 to 125.6	ns	0.4274
BML 2013 vs. BML 2017	122.8	-182.8 to 428.4	ns	0.7288
BML 2013 vs. BML 2015	44.47	-261.2 to 350.1	ns	0.982

Tukey's multiple comparisons test	Mean Diff.	95.00% CI of diff.	Summary	Adjusted P Value
BML 2017 vs. BML 2015	-78.33	-384.0 to 227.3	ns	0.9119
13				
Pond 9 2016 vs. BML 2013	-239.4	-545.1 to 66.20	ns	0.1823
Pond 9 2016 vs. BML 2017	-163.7	-469.4 to 141.9	ns	0.5122
Pond 9 2016 vs. BML 2015	-200.4	-506.1 to 105.2	ns	0.33
BML 2013 vs. BML 2017	75.7	-229.9 to 381.3	ns	0.9196
BML 2013 vs. BML 2015	39	-266.6 to 344.6	ns	0.9877
BML 2017 vs. BML 2015	-36.7	-342.3 to 268.9	ns	0.9897
14				
Pond 9 2016 vs. BML 2013	-260.6	-566.2 to 45.07	ns	0.1253
Pond 9 2016 vs. BML 2017	-207.4	-513.0 to 98.27	ns	0.2998
Pond 9 2016 vs. BML 2015	-223	-528.7 to 82.60	ns	0.2376
BML 2013 vs. BML 2017	53.2	-252.4 to 358.8	ns	0.9699
BML 2013 vs. BML 2015	37.53	-268.1 to 343.2	ns	0.989
BML 2017 vs. BML 2015	-15.67	-321.3 to 290.0	ns	0.9992
15				
Pond 9 2016 vs. BML 2013	-280.3	-585.9 to 25.33	ns	0.0855
Pond 9 2016 vs. BML 2017	-241.2	-546.8 to 64.43	ns	0.1769
Pond 9 2016 vs. BML 2015	-246.3	-552.0 to 59.30	ns	0.162
BML 2013 vs. BML 2017	39.1	-266.5 to 344.7	ns	0.9876
BML 2013 vs. BML 2015	33.97	-271.7 to 339.6	ns	0.9918
BML 2017 vs. BML 2015	-5.133	-310.8 to 300.5	ns	>0.9999
16				
Pond 9 2016 vs. BML 2013	-299.7	-605.4 to 5.900	ns	0.0569
Pond 9 2016 vs. BML 2017	-268.2	-573.9 to 37.40	ns	0.1084
Pond 9 2016 vs. BML 2015	-267.5	-573.2 to 38.10	ns	0.1099
BML 2013 vs. BML 2017	31.5	-274.1 to 337.1	ns	0.9934
BML 2013 vs. BML 2015	32.2	-273.4 to 337.8	ns	0.993
BML 2017 vs. BML 2015	0.7	-304.9 to 306.3	ns	>0.9999
17				
Pond 9 2016 vs. BML 2013	-315.4	-621.0 to -9.733	*	0.0401

Tukey's multiple comparisons test	Mean Diff.	95.00% CI of diff.	Summary	Adjusted P Value
Pond 9 2016 vs. BML 2017	-288.2	-593.8 to 17.43	ns	0.0727
Pond 9 2016 vs. BML 2015	-284.9	-590.5 to 20.77	ns	0.0779
BML 2013 vs. BML 2017	27.17	-278.5 to 332.8	ns	0.9958
BML 2013 vs. BML 2015	30.5	-275.1 to 336.1	ns	0.994
BML 2017 vs. BML 2015	3.333	-302.3 to 309.0	ns	>0.9999
18				
Pond 9 2016 vs. BML 2013	-328.1	-633.7 to -22.43	*	0.0298
Pond 9 2016 vs. BML 2017	-303	-608.7 to 2.600	ns	0.053
Pond 9 2016 vs. BML 2015	-299.4	-605.0 to 6.267	ns	0.0574
BML 2013 vs. BML 2017	25.03	-280.6 to 330.7	ns	0.9967
BML 2013 vs. BML 2015	28.7	-276.9 to 334.3	ns	0.995
BML 2017 vs. BML 2015	3.667	-302.0 to 309.3	ns	>0.9999
19				
Pond 9 2016 vs. BML 2013	-338.9	-644.5 to -33.27	*	0.0229
Pond 9 2016 vs. BML 2017	-314.4	-620.0 to -8.733	*	0.0411
Pond 9 2016 vs. BML 2015	-310.9	-616.6 to -5.300	*	0.0444
BML 2013 vs. BML 2017	24.53	-281.1 to 330.2	ns	0.9969
BML 2013 vs. BML 2015	27.97	-277.7 to 333.6	ns	0.9954
BML 2017 vs. BML 2015	3.433	-302.2 to 309.1	ns	>0.9999
20				
Pond 9 2016 vs. BML 2013	-346.9	-652.5 to -41.27	*	0.0188
Pond 9 2016 vs. BML 2017	-322.2	-627.8 to -16.53	*	0.0343
Pond 9 2016 vs. BML 2015	-318.5	-624.2 to -12.90	*	0.0373
BML 2013 vs. BML 2017	24.73	-280.9 to 330.4	ns	0.9968
BML 2013 vs. BML 2015	28.37	-277.3 to 334.0	ns	0.9952
BML 2017 vs. BML 2015	3.633	-302.0 to 309.3	ns	>0.9999
21				
Pond 9 2016 vs. BML 2013	-351.5	-657.1 to -45.87	*	0.0167
Pond 9 2016 vs. BML 2017	-326.1	-631.7 to -20.43	*	0.0313
Pond 9 2016 vs. BML 2015	-322.3	-628.0 to -16.70	*	0.0342
BML 2013 vs. BML 2017	25.43	-280.2 to 331.1	ns	0.9965
BML 2013 vs. BML 2015	29.17	-276.5 to 334.8	ns	0.9948

Tukey's multiple comparisons test	Mean Diff.	95.00% CI of diff.	Summary	Adjusted P Value
BML 2017 vs. BML 2015	3.733	-301.9 to 309.4	ns	>0.9999
22				
Pond 9 2016 vs. BML 2013	-352.2	-657.8 to -46.53	*	0.0164
Pond 9 2016 vs. BML 2017	-326.4	-632.0 to -20.77	*	0.031
Pond 9 2016 vs. BML 2015	-323.2	-628.8 to -17.57	*	0.0335
BML 2013 vs. BML 2017	25.77	-279.9 to 331.4	ns	0.9964
BML 2013 vs. BML 2015	28.97	-276.7 to 334.6	ns	0.9949
BML 2017 vs. BML 2015	3.2	-302.4 to 308.8	ns	>0.9999
23				
Pond 9 2016 vs. BML 2013	-349.4	-655.0 to -43.77	*	0.0176
Pond 9 2016 vs. BML 2017	-327.3	-632.9 to -21.67	*	0.0304
Pond 9 2016 vs. BML 2015	-322.8	-628.5 to -17.20	*	0.0338
BML 2013 vs. BML 2017	22.1	-283.5 to 327.7	ns	0.9977
BML 2013 vs. BML 2015	26.57	-279.1 to 332.2	ns	0.996
BML 2017 vs. BML 2015	4.467	-301.2 to 310.1	ns	>0.9999
24				
Pond 9 2016 vs. BML 2013	-346.6	-652.3 to -41.00	*	0.0189
Pond 9 2016 vs. BML 2017	-322.3	-628.0 to -16.70	*	0.0342
Pond 9 2016 vs. BML 2015	-320.8	-626.4 to -15.17	*	0.0354
BML 2013 vs. BML 2017	24.3	-281.3 to 329.9	ns	0.997
BML 2013 vs. BML 2015	25.83	-279.8 to 331.5	ns	0.9963
BML 2017 vs. BML 2015	1.533	-304.1 to 307.2	ns	>0.9999
25				
Pond 9 2016 vs. BML 2013	-341.9	-647.6 to -36.30	*	0.0213
Pond 9 2016 vs. BML 2017	-315.9	-621.5 to -10.27	*	0.0397
Pond 9 2016 vs. BML 2015	-316.3	-621.9 to -10.63	*	0.0393
BML 2013 vs. BML 2017	26.03	-279.6 to 331.7	ns	0.9963
BML 2013 vs. BML 2015	25.67	-280.0 to 331.3	ns	0.9964
BML 2017 vs. BML 2015	-0.3667	-306.0 to 305.3	ns	>0.9999
26				
Pond 9 2016 vs. BML 2013	-335.4	-641.1 to -29.80	*	0.025

Tukey's multiple comparisons test	Mean Diff.	95.00% CI of diff.	Summary	Adjusted P Value
Pond 9 2016 vs. BML 2017	-306.9	-612.6 to -1.300	*	0.0486
Pond 9 2016 vs. BML 2015	-309.2	-614.8 to -3.567	*	0.0462
BML 2013 vs. BML 2017	28.5	-277.1 to 334.1	ns	0.9951
BML 2013 vs. BML 2015	26.23	-279.4 to 331.9	ns	0.9962
BML 2017 vs. BML 2015	-2.267	-307.9 to 303.4	ns	>0.9999
27				
Pond 9 2016 vs. BML 2013	-327.8	-633.5 to -22.20	*	0.03
Pond 9 2016 vs. BML 2017	-300.5	-606.1 to 5.167	ns	0.056
Pond 9 2016 vs. BML 2015	-300.1	-605.7 to 5.533	ns	0.0565
BML 2013 vs. BML 2017	27.37	-278.3 to 333.0	ns	0.9957
BML 2013 vs. BML 2015	27.73	-277.9 to 333.4	ns	0.9955
BML 2017 vs. BML 2015	0.3667	-305.3 to 306.0	ns	>0.9999
28				
Pond 9 2016 vs. BML 2013	-318.2	-623.9 to -12.60	*	0.0376
Pond 9 2016 vs. BML 2017	-291.7	-597.3 to 13.97	ns	0.0676
Pond 9 2016 vs. BML 2015	-292.9	-598.5 to 12.73	ns	0.0659
BML 2013 vs. BML 2017	26.57	-279.1 to 332.2	ns	0.996
BML 2013 vs. BML 2015	25.33	-280.3 to 331.0	ns	0.9966
BML 2017 vs. BML 2015	-1.233	-306.9 to 304.4	ns	>0.9999
29				
Pond 9 2016 vs. BML 2013	-309.8	-615.5 to -4.200	*	0.0455
Pond 9 2016 vs. BML 2017	-282.2	-587.8 to 23.43	ns	0.0823
Pond 9 2016 vs. BML 2015	-283.9	-589.5 to 21.73	ns	0.0795
BML 2013 vs. BML 2017	27.63	-278.0 to 333.3	ns	0.9955
BML 2013 vs. BML 2015	25.93	-279.7 to 331.6	ns	0.9963
BML 2017 vs. BML 2015	-1.7	-307.3 to 303.9	ns	>0.9999
30				
Pond 9 2016 vs. BML 2013	-300.6	-606.2 to 5.033	ns	0.0558
Pond 9 2016 vs. BML 2017	-271.2	-576.9 to 34.40	ns	0.1023
Pond 9 2016 vs. BML 2015	-272.8	-578.4 to 32.87	ns	0.0993
BML 2013 vs. BML 2017	29.37	-276.3 to 335.0	ns	0.9947
BML 2013 vs. BML 2015	27.83	-277.8 to 333.5	ns	0.9954

Tukey's multiple comparisons test	Mean Diff.	95.00% CI of diff.	Summary	Adjusted P Value
BML 2017 vs. BML 2015	-1.533	-307.2 to 304.1	ns	>0.9999
31				
Pond 9 2016 vs. BML 2013	-288.7	-594.3 to 16.97	ns	0.072
Pond 9 2016 vs. BML 2017	-259.4	-565.0 to 46.27	ns	0.1282
Pond 9 2016 vs. BML 2015	-260	-565.7 to 45.60	ns	0.1266
BML 2013 vs. BML 2017	29.3	-276.3 to 334.9	ns	0.9947
BML 2013 vs. BML 2015	28.63	-277.0 to 334.3	ns	0.9951
BML 2017 vs. BML 2015	-0.6667	-306.3 to 305.0	ns	>0.9999
32				
Pond 9 2016 vs. BML 2013	-275.6	-581.2 to 30.07	ns	0.094
Pond 9 2016 vs. BML 2017	-245.2	-550.8 to 60.43	ns	0.1652
Pond 9 2016 vs. BML 2015	-245.5	-551.1 to 60.17	ns	0.1644
BML 2013 vs. BML 2017	30.37	-275.3 to 336.0	ns	0.9941
BML 2013 vs. BML 2015	30.1	-275.5 to 335.7	ns	0.9943
BML 2017 vs. BML 2015	-0.2667	-305.9 to 305.4	ns	>0.9999
33				
Pond 9 2016 vs. BML 2013	-261.4	-567.0 to 44.27	ns	0.1235
Pond 9 2016 vs. BML 2017	-231	-536.6 to 74.67	ns	0.2096
Pond 9 2016 vs. BML 2015	-231.8	-537.5 to 73.80	ns	0.2067
BML 2013 vs. BML 2017	30.4	-275.2 to 336.0	ns	0.9941
BML 2013 vs. BML 2015	29.53	-276.1 to 335.2	ns	0.9946
BML 2017 vs. BML 2015	-0.8667	-306.5 to 304.8	ns	>0.9999
34				
Pond 9 2016 vs. BML 2013	-245.6	-551.2 to 60.03	ns	0.164
Pond 9 2016 vs. BML 2017	-215.7	-521.4 to 89.90	ns	0.2655
Pond 9 2016 vs. BML 2015	-216.5	-522.1 to 89.13	ns	0.2625
BML 2013 vs. BML 2017	29.87	-275.8 to 335.5	ns	0.9944
BML 2013 vs. BML 2015	29.1	-276.5 to 334.7	ns	0.9948
BML 2017 vs. BML 2015	-0.7667	-306.4 to 304.9	ns	>0.9999
35				
Pond 9 2016 vs. BML 2013	-228.8	-534.5 to 76.80	ns	0.2169

Tukey's multiple comparisons test	Mean Diff.	95.00% CI of diff.	Summary	Adjusted P Value
Pond 9 2016 vs. BML 2017	-199.5	-505.2 to 106.1	ns	0.334
Pond 9 2016 vs. BML 2015	-199.6	-505.3 to 106.0	ns	0.3336
BML 2013 vs. BML 2017	29.3	-276.3 to 334.9	ns	0.9947
BML 2013 vs. BML 2015	29.2	-276.4 to 334.8	ns	0.9948
BML 2017 vs. BML 2015	-0.1	-305.7 to 305.5	ns	>0.9999
36				
Pond 9 2016 vs. BML 2013	-211.4	-517.1 to 94.20	ns	0.2828
Pond 9 2016 vs. BML 2017	-182	-487.6 to 123.6	ns	0.4176
Pond 9 2016 vs. BML 2015	-182	-487.7 to 123.6	ns	0.4174
BML 2013 vs. BML 2017	29.43	-276.2 to 335.1	ns	0.9946
BML 2013 vs. BML 2015	29.4	-276.2 to 335.0	ns	0.9946
BML 2017 vs. BML 2015	-0.03333	-305.7 to 305.6	ns	>0.9999
37				
Pond 9 2016 vs. BML 2013	-192.7	-498.3 to 112.9	ns	0.3655
Pond 9 2016 vs. BML 2017	-162.9	-468.5 to 142.8	ns	0.5168
Pond 9 2016 vs. BML 2015	-162.6	-468.2 to 143.0	ns	0.5182
BML 2013 vs. BML 2017	29.83	-275.8 to 335.5	ns	0.9944
BML 2013 vs. BML 2015	30.1	-275.5 to 335.7	ns	0.9943
BML 2017 vs. BML 2015	0.2667	-305.4 to 305.9	ns	>0.9999
38				
Pond 9 2016 vs. BML 2013	-173.3	-478.9 to 132.4	ns	0.4621
Pond 9 2016 vs. BML 2017	-142.8	-448.5 to 162.8	ns	0.6244
Pond 9 2016 vs. BML 2015	-142.3	-447.9 to 163.3	ns	0.6273
BML 2013 vs. BML 2017	30.43	-275.2 to 336.1	ns	0.9941
BML 2013 vs. BML 2015	30.97	-274.7 to 336.6	ns	0.9938
BML 2017 vs. BML 2015	0.5333	-305.1 to 306.2	ns	>0.9999
39				
Pond 9 2016 vs. BML 2013	-151.8	-457.5 to 153.8	ns	0.576
Pond 9 2016 vs. BML 2017	-122.8	-428.4 to 182.9	ns	0.7289
Pond 9 2016 vs. BML 2015	-121.5	-427.2 to 184.1	ns	0.7351
BML 2013 vs. BML 2017	29.07	-276.6 to 334.7	ns	0.9948
BML 2013 vs. BML 2015	30.3	-275.3 to 335.9	ns	0.9941

Tukey's multiple comparisons test	Mean Diff.	95.00% CI of diff.	Summary	Adjusted P Value
BML 2017 vs. BML 2015	1.233	-304.4 to 306.9	ns	>0.9999
40				
Pond 9 2016 vs. BML 2013	-129.3	-435.0 to 176.3	ns	0.6956
Pond 9 2016 vs. BML 2017	-101	-406.6 to 204.7	ns	0.8298
Pond 9 2016 vs. BML 2015	-98.47	-404.1 to 207.2	ns	0.8401
BML 2013 vs. BML 2017	28.37	-277.3 to 334.0	ns	0.9952
BML 2013 vs. BML 2015	30.87	-274.8 to 336.5	ns	0.9938
BML 2017 vs. BML 2015	2.5	-303.1 to 308.1	ns	>0.9999
41				
Pond 9 2016 vs. BML 2013	-104.7	-410.4 to 200.9	ns	0.8137
Pond 9 2016 vs. BML 2017	-76.67	-382.3 to 229.0	ns	0.9168
Pond 9 2016 vs. BML 2015	-74.03	-379.7 to 231.6	ns	0.9243
BML 2013 vs. BML 2017	28.07	-277.6 to 333.7	ns	0.9953
BML 2013 vs. BML 2015	30.7	-274.9 to 336.3	ns	0.9939
BML 2017 vs. BML 2015	2.633	-303.0 to 308.3	ns	>0.9999
42				
Pond 9 2016 vs. BML 2013	-79.83	-385.5 to 225.8	ns	0.9073
Pond 9 2016 vs. BML 2017	-52.97	-358.6 to 252.7	ns	0.9703
Pond 9 2016 vs. BML 2015	-49.13	-354.8 to 256.5	ns	0.976
BML 2013 vs. BML 2017	26.87	-278.8 to 332.5	ns	0.9959
BML 2013 vs. BML 2015	30.7	-274.9 to 336.3	ns	0.9939
BML 2017 vs. BML 2015	3.833	-301.8 to 309.5	ns	>0.9999
43				
Pond 9 2016 vs. BML 2013	-53.9	-359.5 to 251.7	ns	0.9688
Pond 9 2016 vs. BML 2017	-27.33	-333.0 to 278.3	ns	0.9957
Pond 9 2016 vs. BML 2015	-22.7	-328.3 to 282.9	ns	0.9975
BML 2013 vs. BML 2017	26.57	-279.1 to 332.2	ns	0.996
BML 2013 vs. BML 2015	31.2	-274.4 to 336.8	ns	0.9936
BML 2017 vs. BML 2015	4.633	-301.0 to 310.3	ns	>0.9999
44				
Pond 9 2016 vs. BML 2013	-26.9	-332.5 to 278.7	ns	0.9959

Tukey's multiple comparisons test	Mean Diff.	95.00% CI of diff.	Summary	Adjusted P Value
Pond 9 2016 vs. BML 2017	-0.3333	-306.0 to 305.3	ns	>0.9999
Pond 9 2016 vs. BML 2015	3.3	-302.3 to 308.9	ns	>0.9999
BML 2013 vs. BML 2017	26.57	-279.1 to 332.2	ns	0.996
BML 2013 vs. BML 2015	30.2	-275.4 to 335.8	ns	0.9942
BML 2017 vs. BML 2015	3.633	-302.0 to 309.3	ns	>0.9999
45				
Pond 9 2016 vs. BML 2013	-0.5	-306.1 to 305.1	ns	>0.9999
Pond 9 2016 vs. BML 2017	25.03	-280.6 to 330.7	ns	0.9967
Pond 9 2016 vs. BML 2015	29.1	-276.5 to 334.7	ns	0.9948
BML 2013 vs. BML 2017	25.53	-280.1 to 331.2	ns	0.9965
BML 2013 vs. BML 2015	29.6	-276.0 to 335.2	ns	0.9945
BML 2017 vs. BML 2015	4.067	-301.6 to 309.7	ns	>0.9999
46				
Pond 9 2016 vs. BML 2013	27.6	-278.0 to 333.2	ns	0.9956
Pond 9 2016 vs. BML 2017	52.03	-253.6 to 357.7	ns	0.9717
Pond 9 2016 vs. BML 2015	58.1	-247.5 to 363.7	ns	0.9614
BML 2013 vs. BML 2017	24.43	-281.2 to 330.1	ns	0.9969
BML 2013 vs. BML 2015	30.5	-275.1 to 336.1	ns	0.994
BML 2017 vs. BML 2015	6.067	-299.6 to 311.7	ns	>0.9999
47				
Pond 9 2016 vs. BML 2013	62.77	-242.9 to 368.4	ns	0.952
Pond 9 2016 vs. BML 2017	87.33	-218.3 to 393.0	ns	0.8824
Pond 9 2016 vs. BML 2015	94.5	-211.1 to 400.1	ns	0.8559
BML 2013 vs. BML 2017	24.57	-281.1 to 330.2	ns	0.9969
BML 2013 vs. BML 2015	31.73	-273.9 to 337.4	ns	0.9933
BML 2017 vs. BML 2015	7.167	-298.5 to 312.8	ns	>0.9999
48				
Pond 9 2016 vs. BML 2013	87.5	-218.1 to 393.1	ns	0.8819
Pond 9 2016 vs. BML 2017	110.6	-195.0 to 416.3	ns	0.7873
Pond 9 2016 vs. BML 2015	118.3	-187.3 to 424.0	ns	0.7508
BML 2013 vs. BML 2017	23.13	-282.5 to 328.8	ns	0.9974
BML 2013 vs. BML 2015	30.83	-274.8 to 336.5	ns	0.9938

Tukey's multiple comparisons test	Mean Diff.	95.00% CI of diff.	Summary	Adjusted P Value
BML 2017 vs. BML 2015	7.7	-297.9 to 313.3	ns	>0.9999
49				
Pond 9 2016 vs. BML 2013	112.1	-193.5 to 417.7	ns	0.7805
Pond 9 2016 vs. BML 2017	134.3	-171.4 to 439.9	ns	0.6699
Pond 9 2016 vs. BML 2015	140.9	-164.8 to 446.5	ns	0.6349
BML 2013 vs. BML 2017	22.17	-283.5 to 327.8	ns	0.9977
BML 2013 vs. BML 2015	28.77	-276.9 to 334.4	ns	0.995
BML 2017 vs. BML 2015	6.6	-299.0 to 312.2	ns	>0.9999
50				
Pond 9 2016 vs. BML 2013	142.8	-162.8 to 448.4	ns	0.6246
Pond 9 2016 vs. BML 2017	163.3	-142.4 to 468.9	ns	0.5147
Pond 9 2016 vs. BML 2015	172	-133.6 to 477.6	ns	0.4686
BML 2013 vs. BML 2017	20.47	-285.2 to 326.1	ns	0.9982
BML 2013 vs. BML 2015	29.2	-276.4 to 334.8	ns	0.9948
BML 2017 vs. BML 2015	8.733	-296.9 to 314.4	ns	0.9999
51				
Pond 9 2016 vs. BML 2013	180.5	-125.1 to 486.1	ns	0.4251
Pond 9 2016 vs. BML 2017	201.7	-103.9 to 507.3	ns	0.3243
Pond 9 2016 vs. BML 2015	212.7	-92.93 to 518.3	ns	0.2776
BML 2013 vs. BML 2017	21.2	-284.4 to 326.8	ns	0.998
BML 2013 vs. BML 2015	32.2	-273.4 to 337.8	ns	0.993
BML 2017 vs. BML 2015	11	-294.6 to 316.6	ns	0.9997
52				
Pond 9 2016 vs. BML 2013	212	-93.63 to 517.6	ns	0.2805
Pond 9 2016 vs. BML 2017	232.9	-72.73 to 538.5	ns	0.2032
Pond 9 2016 vs. BML 2015	242.3	-63.37 to 547.9	ns	0.1737
BML 2013 vs. BML 2017	20.9	-284.7 to 326.5	ns	0.9981
BML 2013 vs. BML 2015	30.27	-275.4 to 335.9	ns	0.9942
BML 2017 vs. BML 2015	9.367	-296.3 to 315.0	ns	0.9998
53				
Pond 9 2016 vs. BML 2013	248.2	-57.47 to 553.8	ns	0.1568

Tukey's multiple comparisons test	Mean Diff.	95.00% CI of diff.	Summary	Adjusted P Value
Pond 9 2016 vs. BML 2017	268.6	-37.00 to 574.3	ns	0.1076
Pond 9 2016 vs. BML 2015	278.2	-27.47 to 583.8	ns	0.0892
BML 2013 vs. BML 2017	20.47	-285.2 to 326.1	ns	0.9982
BML 2013 vs. BML 2015	30	-275.6 to 335.6	ns	0.9943
BML 2017 vs. BML 2015	9.533	-296.1 to 315.2	ns	0.9998
54				
Pond 9 2016 vs. BML 2013	292	-13.63 to 597.6	ns	0.0672
Pond 9 2016 vs. BML 2017	310.9	5.267 to 616.5	*	0.0444
Pond 9 2016 vs. BML 2015	321.8	16.20 to 627.5	*	0.0346
BML 2013 vs. BML 2017	18.9	-286.7 to 324.5	ns	0.9986
BML 2013 vs. BML 2015	29.83	-275.8 to 335.5	ns	0.9944
BML 2017 vs. BML 2015	10.93	-294.7 to 316.6	ns	0.9997
55				
Pond 9 2016 vs. BML 2013	336.8	31.17 to 642.4	*	0.0241
Pond 9 2016 vs. BML 2017	355.8	50.17 to 661.4	*	0.0149
Pond 9 2016 vs. BML 2015	365.4	59.77 to 671.0	*	0.0116
BML 2013 vs. BML 2017	19	-286.6 to 324.6	ns	0.9985
BML 2013 vs. BML 2015	28.6	-277.0 to 334.2	ns	0.9951
BML 2017 vs. BML 2015	9.6	-296.0 to 315.2	ns	0.9998
56				
Pond 9 2016 vs. BML 2013	379.6	74.00 to 685.3	**	0.0079
Pond 9 2016 vs. BML 2017	399	93.37 to 704.6	**	0.0045
Pond 9 2016 vs. BML 2015	407.9	102.2 to 713.5	**	0.0035
BML 2013 vs. BML 2017	19.37	-286.3 to 325.0	ns	0.9985
BML 2013 vs. BML 2015	28.23	-277.4 to 333.9	ns	0.9953
BML 2017 vs. BML 2015	8.867	-296.8 to 314.5	ns	0.9999
57				
Pond 9 2016 vs. BML 2013	434.9	129.2 to 740.5	**	0.0015
Pond 9 2016 vs. BML 2017	452.8	147.1 to 758.4	***	0.0009
Pond 9 2016 vs. BML 2015	461.9	156.3 to 767.5	***	0.0006
BML 2013 vs. BML 2017	17.9	-287.7 to 323.5	ns	0.9988
BML 2013 vs. BML 2015	27.03	-278.6 to 332.7	ns	0.9958

Tukey's multiple comparisons test	Mean Diff.	95.00% CI of diff.	Summary	Adjusted P Value
BML 2017 vs. BML 2015	9.133	-296.5 to 314.8	ns	0.9998
58				
Pond 9 2016 vs. BML 2013	485.7	180.1 to 791.3	***	0.0003
Pond 9 2016 vs. BML 2017	502.6	197.0 to 808.3	***	0.0002
Pond 9 2016 vs. BML 2015	512.6	207.0 to 818.3	***	0.0001
BML 2013 vs. BML 2017	16.93	-288.7 to 322.6	ns	0.999
BML 2013 vs. BML 2015	26.93	-278.7 to 332.6	ns	0.9959
BML 2017 vs. BML 2015	10	-295.6 to 315.6	ns	0.9998
59				
Pond 9 2016 vs. BML 2013	544.3	238.7 to 850.0	****	<0.0001
Pond 9 2016 vs. BML 2017	560.7	255.1 to 866.4	****	<0.0001
Pond 9 2016 vs. BML 2015	570.6	265.0 to 876.3	****	<0.0001
BML 2013 vs. BML 2017	16.4	-289.2 to 322.0	ns	0.9991
BML 2013 vs. BML 2015	26.3	-279.3 to 331.9	ns	0.9962
BML 2017 vs. BML 2015	9.9	-295.7 to 315.5	ns	0.9998
60				
Pond 9 2016 vs. BML 2013	600.6	294.9 to 906.2	****	<0.0001
Pond 9 2016 vs. BML 2017	616.5	310.8 to 922.1	****	<0.0001
Pond 9 2016 vs. BML 2015	626.2	320.6 to 931.8	****	<0.0001
BML 2013 vs. BML 2017	15.9	-289.7 to 321.5	ns	0.9991
BML 2013 vs. BML 2015	25.63	-280.0 to 331.3	ns	0.9964
BML 2017 vs. BML 2015	9.733	-295.9 to 315.4	ns	0.9998
